# Survey of High Throughput RNA-Seq Data Reveals Potential Roles for lncRNAs during Development and Stress Response in Bread Wheat

**DOI:** 10.3389/fpls.2017.01019

**Published:** 2017-06-09

**Authors:** Shumayla, Shailesh Sharma, Mehak Taneja, Shivi Tyagi, Kashmir Singh, Santosh K. Upadhyay

**Affiliations:** ^1^Department of Botany, Panjab UniversityChandigarh, India; ^2^National Agri-Food Biotechnology InstituteMohali, India; ^3^Department of Biotechnology, Panjab UniversityChandigarh, India

**Keywords:** bread wheat, co-expression, developmental process, gene ontology, lncRNA, stress response, miRNA

## Abstract

Long non-coding RNAs (lncRNAs) are a family of regulatory RNAs that play essential role in the various developmental processes and stress responses. Recent advances in sequencing technology and computational methods enabled identification and characterization of lncRNAs in certain plant species, but they are less known in *Triticum aestivum* (bread wheat). Herein, we analyzed 52 RNA seq data (>30 billion reads) and identified 44,698 lncRNAs in *T. aestivum* genome, which were characterized in comparison to the coding sequences (mRNAs). Similar to the mRNAs, lncRNAs were also derived from each sub-genome and chromosome, and showed tissue developmental stage specific and differential expression, as well. The modulated expression of lncRNAs during abiotic stresses like heat, drought, and salt indicated their putative role in stress response. The co-expression of lncRNAs with vital mRNAs including various transcription factors and enzymes involved in Abscisic acid (ABA) biosynthesis, and gene ontology mapping inferred their regulatory roles in numerous biological processes. A few lncRNAs were predicted as precursor (19 lncRNAs), while some as target mimics (1,047 lncRNAs) of known miRNAs involved in various regulatory functions. The results suggested numerous functions of lncRNAs in *T. aestivum*, and unfolded the opportunities for functional characterization of individual lncRNA in future studies.

## Introduction

About 90% of the genome of an organism is transcribed into RNAs, but majority of them are non-coding (Ariel et al., 2015). These non-coding RNAs (ncRNAs) are crucial for gene functioning, and cover both housekeeping as well as regulatory ncRNAs (Cech and Steitz, 2014). The housekeeping ncRNAs (e.g., t-RNA and r-RNA) express in all cell types, while the regulatory ncRNAs are usually specific to different cell types. The regulatory ncRNAs consist of small ncRNAs (i.e., miRNAs and siRNAs) and long ncRNAs (Kim and Sung, 2012; Liu et al., 2012). The lncRNAs are key regulatory elements, involved in numerous developmental processes and stress responses (Kim and Sung, 2012). These are usually ≥200 bp in length, which lack coding potential. They may be nuclear or cytoplasmic (Liu J. et al., 2015). They share similarity with mRNA in many aspects like splicing, polyadenylation, and conserved sequences (Guttman et al., 2009; Khalil et al., 2009). They are also transcribed by RNA polymerase II like mRNA (Xin et al., 2011). Moreover, two plant specific RNA polymerases (RNA Pol IV and Pol V) evolved from RNA polymerases II, trigger lncRNA transcription. These polymerases are required for regulation of gene expression by gene silencing mechanism and epigenetic control (Wierzbicki et al., 2008). The polyadenylation is required for stability and nuclear export of RNA. The lncRNAs can be polyadenylated or non-polyadenylated. The non-polyadenylated lncRNAs are usually shorter and exhibit lower expression, but they are more specific to stress response than the polyadenylated lncRNAs (Yang et al., 2011; Di et al., 2014). The lncRNAs can transcribe from any position of a genome. They can be intronic, intergenic, and/or overlapping with intron or exon of coding genes. They can be formed in both sense and anti-sense direction. Natural anti-sense RNAs (NATs) are a class of lncRNA that represent partial or complete base complementation to the coding transcripts. They may act as *cis*- or *trans*- regulatory elements and regulate gene transcription during tissue development and stress conditions (Kim and Sung, 2012).

The lncRNAs have been extensively investigated in the animals, and found to be involved in numerous cellular processes ranging from cell-cycle regulation, pluripotency to cancer (Rinn and Chang, 2012), but they are comparatively less studied in plants. The function of most of the lncRNAs is still unknown in plants, but the available reports indicate their role in various stress conditions and biological processes. A few plant species including Arabidopsis, cucumber, maize, poplar, and rice have been explored to understand the role and mechanism of lncRNAs (Ding et al., 2012; Li L. et al., 2014; Shuai et al., 2014; Wunderlich et al., 2014; Zhang Y. et al., 2014; Hao Z. et al., 2015; Bhatia et al., 2017). In Arabidopsis, *COOLAIR* and *COLDAIR* lncRNAs regulate flowering through promoter interference and histone modification, respectively (Wunderlich et al., 2014). Role of lncRNAs in flower development has also been suggested in chickpea (Khemka et al., 2016). In rice, *LDMAR* (long day specific male-fertility-associated RNA) is involved in male sterility (Ding et al., 2012). Nodulation in soybean, rice, and *Medicago truncatula* are dependent upon *GmENOD40, OsENOD4*, and *MtENOD40* lncRNAs, respectively (Yang et al., 1993; Kouchi et al., 1999; Sousa et al., 2001). Furthermore, the role of lncRNAs in wood formation in poplar, photomorphogenesis in Arabidopsis, and phosphate homeostasis in Arabidopsis, tomato and *M. truncatula* have been identified (Franco-Zorrilla et al., 2007; Rymarquis et al., 2008; Wang et al., 2014).

The lncRNAs also regulate the miRNA functioning by acting as target mimics or decoy in both plants and animals. They inhibit the interaction between miRNAs and its target mRNAs (Franco-Zorrilla et al., 2007; Wu et al., 2013). In plants, lncRNA *Induced by Phosphate Starvation 1* (*IPS1*) which interferes in binding of ath-miR399 to its specific target, was first discovered in Arabidopsis (Franco-Zorrilla et al., 2007). Later on, the target mimicry was further identified in other plant species (Ivashuta et al., 2011; Khemka et al., 2016). Artificial target mimicry can be introduced in the transgenic plant to alter the function of corresponding miRNA (Ivashuta et al., 2011). Besides this, lncRNA also act as translational enhancer in rice during phosphate homeostasis, alternative splicing regulators in Arabidopsis resulting in lateral root development, and cause degradation of dsRNA for local cytokinin synthesis in *Petunia* (Zubko and Meyer, 2007; Jabnoune et al., 2013; Bardou et al., 2014). Stress responsive lncRNAs under drought, cold, salt, abscisic acid, and Ef-Tu treatment (to induce biotic stress) have been identified in Arabidopsis (Liu et al., 2012). These findings indicate the regulatory role of lncRNAs in diverse biological processes.

The high-throughput RNA sequence (RNA seq) data produced by next-generation sequencing have decrypted the prominence of earlier known “junk DNA” including non-coding transcripts in both plant and animal species (Liu J. et al., 2015). A few fungal and heat stress responsive lncRNAs are also reported in *Triticum aestivum* (Xin et al., 2011; Zhang H. et al., 2016), but the analyses were performed with either microarray data or very limited set of RNA seq data. Since the *T. aestivum* is an important and widely grown crop plant, it is necessary to characterize each aspect in great detail including the role of lncRNAs. Further, the availability of genomic and in-depth transcriptomic information of *T. aestivum* in recent years enabled the characterization of numerous aspects at genome scale (IWGSC, 2014; Liu Z. et al., 2015; Pingault et al., 2015; Zhang Y. et al., 2016). Here, we decoded the lncRNAs of *T. aestivum* using 52 high-throughput RNA seq data generated from three developmental stages of five tissues, and heat, drought, and salt stress treatments, as well. However, we could not classify them in various categories like sense, anti-sense, intronic, intergenic, or NATs, due to unavailability of the complete genome sequence. The identified lncRNAs were characterized in comparison to the coding sequences (mRNA) of *T. aestivum* (IWGSC, 2014). The functional annotation of lncRNAs was carried out by co-expression analysis with mRNAs and gene ontology (GO) mapping, which predicted their role in numerous biological processes. The lncRNAs were also analyzed for their role during development and various abiotic stress conditions by expression profiling, co-expression and GO analysis in comparison to the mRNAs. The tissue specific expression of certain lncRNAs hinted at their role in development of related tissue, while modulated expression under stress treatments suggested their stress responsive function. Further, the regulatory role of lncRNAs was analyzed by their co-expression analysis with various transcription factors (TFs), and enzymes involved in Abscisic acid (ABA) biosynthesis. The miRNAs mediated interaction of lncRNAs with mRNAs was also analyzed. The lncRNAs could act as both precursor and target mimic of miRNA in *T. aestivum*. These interactions refine the idea about the mechanism of action of lncRNAs in *T. aestivum*.

## Materials and methods

### Data sets used for the identification of lncRNAs

A total of 52 high throughput RNA seq data were surveyed for the identification of lncRNAs in *T. aestivum* (File S1). These RNA seq data were generated in replicates from five different tissue samples (root, stem, leaf, spike, and grain) at three different developmental stages, and after various abiotic stress (heat, drought, and salt) treatments (Liu Z. et al., 2015; Pingault et al., 2015; Zhang Y. et al., 2016). The data for tissue developmental stages are available at https://urgi.versailles.inra.fr/Files/RNASeqWheat/, whereas for abiotic stress at NCBI SRA database (https://www.ncbi.nlm.nih.gov/sra) with accession numbers SRP045409 and SRP062745.

### Bioinformatics pipeline for isolation and characterization of lncRNA

**Figure 1:**
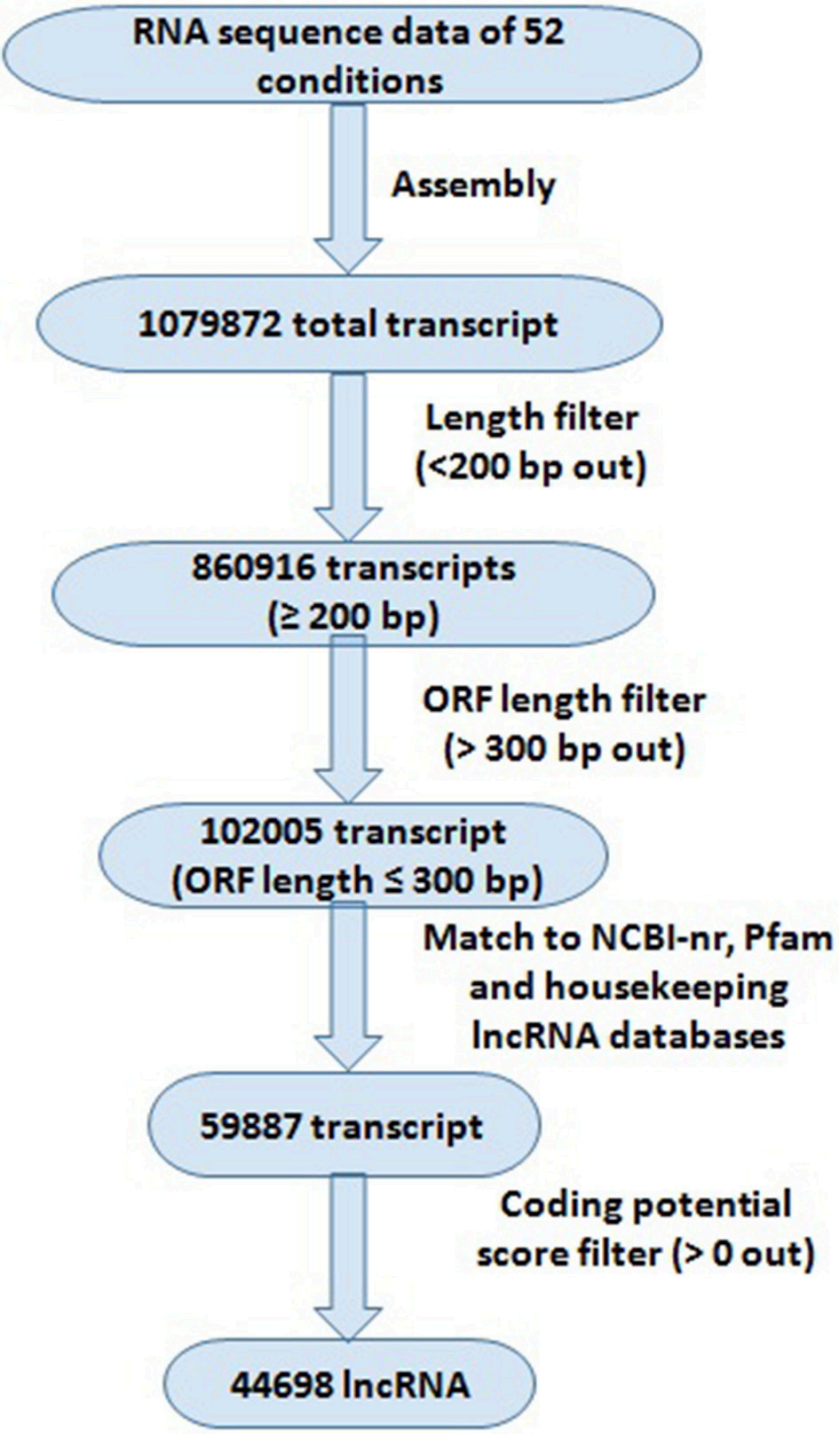
Systematic representation of bioinformatics approach for the identification of lncRNAs in *Triticum aestivum*. Different filters were applied for the identification of lncRNAs; numbers representing total number of transcripts identified at each filter.

The pipeline used for the identification of lncRNA has been described in Figure 1. The RNA seq data were quality filtered using SRA toolkit (https://www.ncbi.nlm.nih.gov/sra/docs/toolkitsoft/) and assembled using Trinity package following the standard procedure (Haas et al., 2013). First the individual SRA reads were assembled separately, and then merged together. The transcripts shorter than 200 bp length were discarded. The open reading frames (ORFs) of filtered transcripts were analyzed using Orf Predictor (Min et al., 2005) web server (http://proteomics.ysu.edu/tools/OrfPredictor.html) and the transcripts with >300 bp ORF lengths were discarded. The filtered sequences were used for BLAST search against NCBI-nr protein and PFam (Finn et al., 2014) databases with *e*-value 10^−3^ to remove the transcripts matched to any reported protein and protein family domain. The remaining transcripts were subjected to coding potential calculation using coding potential calculator (http://cpc.cbi.pku.edu.cn) following the standard procedure (Kong et al., 2007). The transcripts having more than 0 coding potential score were discarded. The housekeeping genes (including tRNAs, rRNAs, snRNAs, and snoRNAs) were extracted by aligning the lncRNA to the housekeeping lncRNA databases (http://gtrnadb2009.ucsc.edu/, http://www.plantrdnadatabase.com/, http://noncode.org/).

To obtain the chromosomal localization of identified lncRNAs, the available chromosome sequences of *T. aestivum* were downloaded from Ensembl Plants and URGI servers (http://plants.ensembl.org/Triticum_aestivum/; https://urgi.versailles.inra.fr/) and BLASTn (*e*-value 10^−10^) search was performed.

### Expression analysis

The expression profile of lncRNA and mRNA sequences (IWGSC, 2014) of *T. aestivum* was studied in various tissues developmental stages and abiotic stress conditions. The tissue specific expression analysis was carried out using high throughput RNA-seq data (https://urgi.versailles.inra.fr/Files/RNASeqWheat/) generated from three developmental stages of root, stem, leaf, spike, and grain in duplicates (Pingault et al., 2015). The expression value was calculated in terms of fragments per kilobase of transcript per million fragments mapped (FPKM) using RNA-Seq by Expectation-Maximization (RSEM) method (Li and Dewey, 2011) from Trinity package (Haas et al., 2013).

The expression analysis was also carried out under various abiotic stresses (heat, drought and salt). The RNA-seq data (accession number: SRP045409) generated in duplicate from leaves samples after 1 and 6 h of incubation under heat (HS), drought (DS), and their combination (HD) were used (Liu Z. et al., 2015). The effect of salt stress was analyzed using root RNA-seq data (accession number: SRP062745) developed after 6, 12, 24, and 48 h of treatment (Zhang Y. et al., 2016). The FPKM-value was calculated as mentioned above, and differential expression was analyzed using EdgeR (Robinson et al., 2010) of Trinity tool (Haas et al., 2013). The comparative expression analysis of lncRNA and mRNA transcripts of *T. aestivum* was analyzed and heat maps for various expression data were generated using Hierarchical Clustering Explorer 3.5 (http://www.cs.umd.edu/hcil/hce/).

### Co-expression and gene ontology (GO) enrichment analysis

The functional annotation of identified lncRNAs was performed using co-expression analysis and gene ontology (GO) mapping with coding mRNA sequences of *T. aestivum*, as reported in earlier studies (Liao et al., 2011; Mattick and Rinn, 2015; Wu et al., 2016). The expression value (FPKM) of each lncRNA and mRNA was calculated in various tissue developmental stages and stress conditions using Trinity as described above. These expression data were used for co-expression analysis using CoExpress v.1.5 tool following the protocol provided in user manual (Nazarov et al., 2013). The lncRNA and mRNA transcripts having maximum expression value <10 FPKM were not used for co-expression analysis. The co-expressed lncRNA/mRNA pair was determined using Pearson correlation coefficient with correlation power 1 and threshold filter 0.9. The GO analysis of mRNA was performed using agriGO tool (http://bioinfo.cau.edu.cn/agriGO/index.php) with fisher statistics (Du et al., 2010). The GO terms obtained for individual mRNA with *p*-value < e^−10^ were used for the functional annotation of co-expressed lncRNAs. The GO terms were further analyzed using REVIGO web server (http://revigo.irb.hr/), which summarizes the results by removing redundant GO terms and provides graph-based visualization (Supek et al., 2011).

The co-expression and GO enrichment analyses were carried out for lncRNAs showing high expression in various tissues, and differential expression during abiotic stresses following above mentioned statistics, separately. The lncRNAs having expression value >10 FPKM in at least one stage were considered for the analysis. In case of abiotic stresses, the analysis was performed for the lncRNAs having ≥5-fold differential expression.

The co-expression analysis was also performed with known TFs of *T. aestivum* available at Plant Transcription Factor Database (http://planttfdb.cbi.pku.edu.cn/, Jin et al., 2017). Further, to reveal the role of lncRNAs in ABA biosynthesis, the orthologous sequences of four important enzymes [β-carotene hydroxylase (BCH), zeaxanthin epoxidase (ZEP), 9-cis-epoxycarotenoid dioxygenase (NCED), and abscisic aldehyde oxidase (AAO)] from *T. aestivum* were obtained by BLAST search of known sequences from Arabidopsis (Finkelstein, 2013), and used for co-expression analysis. The Arabidopsis sequences used for BLAST search were- AT4G25700 and AT5G52570 for BCH, AT5G67030 for ZEP, AT4G18350, AT3G14440, AT1G30100, and AT1G78390 for NCED, and AT2G27150 for AAO.

### Calculation of developmental stage specificity index

To elucidate the tissue developmental stage specific expression of lncRNAs, the specificity index (SI) was calculated following the method described (Julien et al., 2012; Kryuchkova-Mostacci and Robinson-Rechavi, 2016). The SI-value of individual gene in each developmental stage was calculated by dividing their consensus expression value by their whole consensus expression value. The SI-value ranged from 0 to 1 for housekeeping to developmental stage specific, respectively. The lncRNAs having SI threshold >0.75 were considered as stage specific.

### Interaction analyses of lncRNAs and/or mRNAs with miRNAs

A total of 607 known miRNA sequences of *T. aestivum* (Sun et al., 2014) were utilized for interaction analyses with lncRNA and mRNA sequences using psRNATarget (http://plantgrn.noble.org/psRNATarget/) server (Dai and Zhao, 2011). The miRNA and lncRNA and/or mRNA sequences were submitted to server and interaction was analyzed at stringent parameters using maximum expectation 2.0 and target accessibility 25.0. The information regarding the interaction of miRNAs with lncRNAs and/or mRNAs was used for interaction network development using Gephi 0.9.1 (https://gephi.org/) tool (Bastian et al., 2009).

### Identification of lncRNA as precursor of miRNA

To identify the lncRNA functions as precursor of miRNA, the precursor sequences of known 438 miRNAs (Sun et al., 2014) were downloaded and individually aligned with identified lncRNAs. An lncRNA harboring a miRNA precursor sequence with 100% query coverage and similarity was considered as precursor of that miRNA. The hairpin loop formation in lncRNAs was analyzed using miRNAFold server (https://evryrna.ibisc.univ-evry.fr/miRNAFold; Tav et al., 2016), and secondary structure was plotted using Vienna RNAfold web server (http://rna.tbi.univie.ac.at/; Gruber et al., 2015).

## Results and discussion

### Identification of lncRNAs

The high-throughput RNA seq data facilitate the detection of novel transcripts quantitatively. We sought comprehensive identification of lncRNAs involved in development and abiotic stress response in *T. aestivum*. Therefore, *in-total* 52 high-throughput RNA seq data, comprising more than 30 billion high quality reads generated from numerous tissue developmental stages, and under various abiotic stress conditions were utilized (File S1). A standard procedure as reported in earlier studies for the identification of lncRNA in plants (Li J. et al., 2014; Li L. et al., 2014; Khemka et al., 2016) was followed (Figure 1). A total of 1079872 unique set of non-overlapping transcripts were generated after assembly of various RNA seq data, which could be both coding and non-coding. These transcripts were subjected to an optimized pipeline to extract lncRNA transcripts (Figure 1). About 20% (218956) transcripts were discarded due to their length shorter than 200 bp. Further, ~88% (758911) remaining transcripts were discarded, which consisted of an open reading frame (ORF) encoding ≥100 amino acid residues long polypeptide chain. The BLAST search against NCBI-nr protein, Pfam, and housekeeping RNA (tRNAs, snRNAs, and snoRNAs) databases were useful in removing 42,118 transcripts due to their similarity with known proteins and housekeeping RNAs. The remaining 59,887 transcripts were subjected to Coding Potential Calculator (CPC), which further filtered 15,189 transcripts having CPC score more than 0, and ultimately resulted into the identification of 44,698 transcripts as high confidence lncRNAs in *T. aestivum*. The CPC is based on transcripts quality, completeness and ORF homology with the reported proteins in various databases (Kong et al., 2007). Similarly, numerous lncRNAs have been identified in various plant species like Arabidopsis, maize, chickpea, mulberry, tomato etc., (Song et al., 2009; Li L. et al., 2014; Wang et al., 2014; Wang J. et al., 2015; Zhang W. et al., 2014; Khemka et al., 2016). However, the number of lncRNAs identified in *T. aestivum* was much higher than the other plants, which is probably due to- (i) the large (~17 Gb) composite allohexaploid (AABBDD) nature of *T. aestivum* genome (Marcussen et al., 2014), or/and (ii) the number of RNA seq data utilized in present study was more than the earlier studies in other plants. Moreover, higher number of coding transcripts and genes in various gene families is also reported in *T. aestivum* in comparison to the other plant species (IWGSC, 2014; Shumayla et al., 2016a,b; Taneja et al., 2016).

### Genome-wide distribution and characterization of lncRNAs

**Figure 2:**
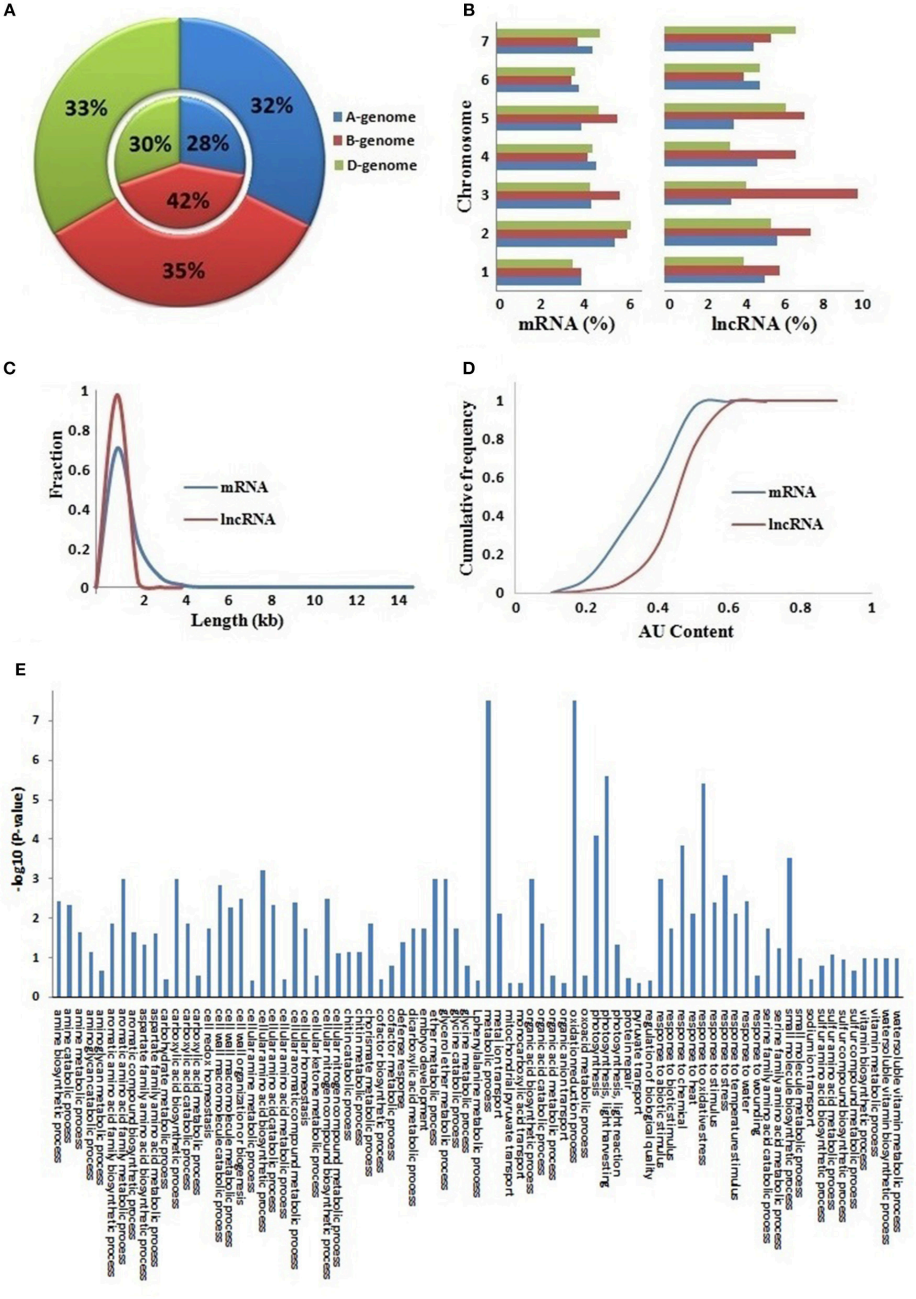
Comparative characteristics analysis of lncRNAs with mRNAs. **(A)** Distribution of lncRNAs and mRNAs on **(A,B** and **D)** sub-genomes. The outer and inner layers represent mRNAs and lncRNAs distribution, respectively. **(B)** Chromosome-wise distribution of lncRNAs and mRNAs. The graph shows length distribution **(C)** and AU content **(D)** of lncRNAs (red) and mRNAs (blue). **(E)** Figure shows gene ontology (GO) enrichment analysis of lncRNAs co-expressed with mRNAs.

The *T. aestivum* genome is derived by the hybridization of three (A, B, and D) subgenomes (Marcussen et al., 2014) which almost equally contributed in composition of various protein coding gene families (IWGSC, 2014; Shumayla et al., 2016a,b; Taneja et al., 2016). Therefore, we analyzed the contribution of these sub-genomes in the composition of lncRNAs (Figures 2A,B, File S2). About 32, 35, and 33% of mRNAs, and 28, 42, and 30% of lncRNAs were located on A, B, and D sub-genomes, respectively. Similarly, these were distributed on each chromosome of various subgenomes but at varied frequency. The majority of chromosomes comprised 3–6% mRNAs and lncRNAs, whereas a few like chromosome 3B consisted of upto 10% lncRNAs. The results established that the lncRNAs are derived from each sub-genome and chromosome of allohexaploid *T. aestivum* genome (Figures 2A,B) as reported in case of various protein coding mRNAs (IWGSC, 2014).

The lncRNAs were further characterized for various important attributes like length and AU content. The length of lncRNAs varied from 200 to 3,570 bp (TR35901) with an average of 453 bp. The average length of lncRNAs was shorter than the average length of coding sequences (832 bp) in *T. aestivum*. Further, the majority of lncRNA (97%) were <1,000 bp in length (Figures 2C, File S2). A total of 11 lncRNAs had length higher than 3,000 bp. The results were in agreement with the earlier reports in other plant species such as Arabidopsis, rice, chickpea, *Ganoderma*, maize, and cucumber, where the mean lncRNA length varied from 322 bp in *Ganoderma* to 800 bp in rice (Li J. et al., 2014; Li L. et al., 2014; Wang et al., 2014; Wang J. et al., 2015; Wang T. et al., 2015; Zhang W. et al., 2014; Zhang Y. et al., 2014; Hao Z. et al., 2015; Khemka et al., 2016).

The AU content of lncRNAs varied from 15 (TR32742) to 93% (TR33790) with an average of 55%. The majority (78%) of lncRNAs comprised more than 50% AU content, in which 29% lncRNAs consisted of more than 60% AU content. A total of 18 lncRNAs had AU content ≥80% (Figures 2D, File S2). However, in case of coding sequences, the AU content varied from 9 to 73% with an average of 46%. The results indicated that the lncRNAs were AU rich as compared to the coding sequences in *T. aestivum* (Figure 2D), as reported in case of various other plant species (Liu et al., 2012; Zhang W. et al., 2014; Zhang Y. et al., 2014; Hao Z. et al., 2015; Khemka et al., 2016).

### Functional annotation

It has been demonstrated in earlier studies that the lncRNAs are involved in numerous biological processes including growth and development to stress response (Ulitsky and Bartel, 2013; Liu J. et al., 2015). Hence, it becomes imperative to analyze the putative function of newly identified lncRNAs. A standard procedure for functional annotation of lncRNAs has still not been established. Further, the annotation of new lncRNAs on the basis of homology with known lncRNAs is also not feasible due to the high sequence divergence among various species. A few methods are recently reported for the annotation of these transcripts, in which co-expression analysis with coding transcripts has been utilized in the majority of studies in both plant and animal species (Liao et al., 2011; Guo et al., 2013; Hao Y. et al., 2015; Mattick and Rinn, 2015; Khemka et al., 2016; Lv et al., 2016; Wu et al., 2016). Here, we followed the similar procedure. Out of 22,549 lncRNAs analyzed having expression value ≥10 FPKM in one or more developmental stages, 7,743 (34%) lncRNAs could be annotated with a putative function (File S3). The functional annotation indicated that the *T. aestivum* lncRNAs may also participate in diverse biological processes as reported in case of other plant species (Li J. et al., 2014; Li L. et al., 2014; Hao Z. et al., 2015; Khemka et al., 2016). The biological processes like photosynthesis, response to different kinds of biotic and abiotic stimulus, organic acid and amino acid metabolism and various other metabolic processes were presumed to be associated with identified lncRNAs (Figure 2E).

### Expression profiling during various tissue developmental stages

**Figure 3:**
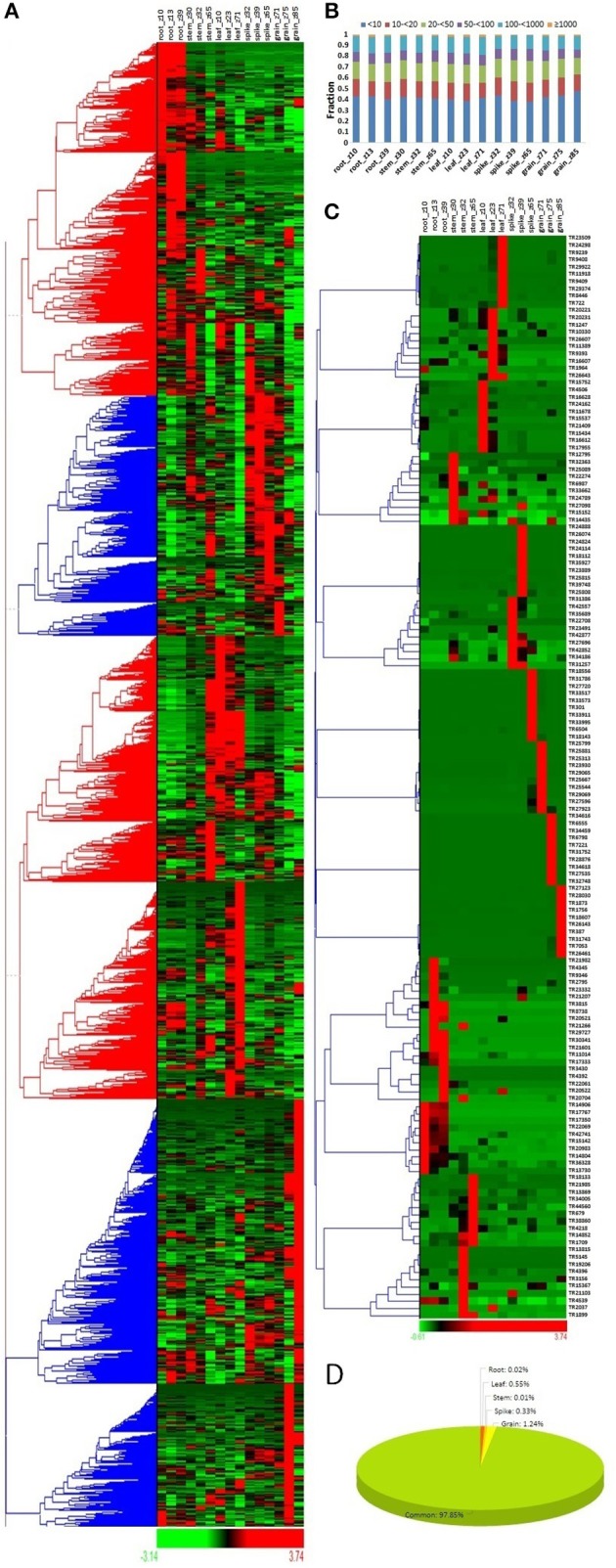
Expression profiling of lncRNA transcripts in various tissue developmental stages. **(A)** Heat map represents the relative expression pattern of 22549 lncRNAs of *T. aestivum* in three developmental stages of five tissues (root, leaf, stem, spike, and grain). The developmental stages are shown in Zadoks scale. **(B)** Distribution of lncRNAs in various categories on the basis of expression level in different developmental stages. **(C)** Heat map shows relative expression profile of top expressing lncRNAs from various developmental stages. **(D)** Pie chart shows percentage of lncRNAs having specific expression in various tissues. Grain showed highest proportion of specifically expressed lncRNAs.

The expression profile of a gene provides insight into their role in various biological functions. To reveal the role of lncRNAs in various tissue developmental stages, their expression analyses were explored in three developmental stages of five different tissues (root, leaf, stem, spike and grain) in comparison to mRNAs of *T. aestivum*. Similar to the mRNAs, the majority of lncRNAs showed differential expression in various tissue developmental stages (Figure 3A, Figure S1A). The results depicted their role in related stages. On the basis of expression value (FPKM), the lncRNAs and mRNAs were divided into six categories- extremely low (<10 FPKM) to extremely high (>1,000 FPKM) expressing. Although significant number of lncRNAs were found to be high expressing (>100 FPKM), the majority were extremely low expressing (<10 FPKM) (Figure 3B). In contrary, about half of the mRNAs were found as high expressing (Figure S1B). Similarly, low expressing lncRNAs were predominantly found in case of other plant species like Arabidopsis, chickpea, cucumber, maize, and rice (Li J. et al., 2014; Li L. et al., 2014; Zhang W. et al., 2014; Zhang Y. et al., 2014; Hao Z. et al., 2015; Khemka et al., 2016). Moreover, the expression value for various categories varied in different plants. It might be due to the differences in genetic background and/or plant species and/or depth of sequencing data utilized for analysis.

The expression profiling of selected 10 highly expressed lncRNAs from each developmental stage indicated that most of them were specific to their related stages (Figure 3C). For instance, lncRNAs TR1756 and TR1873 were found to be specifically expressed in grain_Z85, while TR9409 and TR8446 were specific to leaf_Z71. Similarly, some mRNAs were also found specifically highly expressed in certain tissues (Figure S1C). The results indicated similar expression behavior of lncRNAs to the mRNAs. Further, we observed that these highly expressed transcripts were usually developmental stage specific rather than tissue specific. Therefore, the specificity index (SI) was also calculated for both lncRNA and mRNA transcripts to analyze their specific expression in various tissue developmental stages as reported in earlier studies (Julien et al., 2012; Khemka et al., 2016; Kryuchkova-Mostacci and Robinson-Rechavi, 2016). Out of 22549 expressing lncRNAs, only 485 (~2%) lncRNAs were found as specific to a particular developmental stage as per the criteria followed (Figure 3D, File S4), and majority (279; 1.24%) of them were specific to grain, which was followed by leaf (125; 0.55%). About similar pattern was observed in case of mRNA, where ~3.5% was found specific, in which 1.24, 1.79, and 0.41% mRNAs were specific to grain, spike, and leaf, respectively (Figure S1D). Further analysis indicated that the majority of developmental stage specific lncRNAs were linked to the later developmental stages, such as 135/279, 55/74, and 102/125 lncRNAs were specific to grain_Z85, spike_Z65 and leaf_Z71 stages in these tissues, respectively (File S4). The results indicated the major roles of these specific lncRNAs in later developmental stages. Moreover, the constitutively expressed lncRNAs might play vital function during the other stages. However, it needs to be functionally validated in later studies. Large number of highly expressed lncRNAs are also reported in actively diving cells and reproductive tissues in other plant species (Liu et al., 2012; Li J. et al., 2014; Li L. et al., 2014; Zhang W. et al., 2014; Zhang Y. et al., 2014; Hao Z. et al., 2015; Kang and Liu, 2015; Khemka et al., 2016). Further, stage specific expression of lncRNAs is also detected in chickpea (Khemka et al., 2016).

The tissue developmental specific expression of lncRNAs suggested their specialized role as reported in case of earlier studies. For instance, anther specific lncRNA *Zm401* of maize and shoot specific lncRNA *IPS1* of Arabidopsis were found associated with male sterility and phosphate homeostasis, respectively (Franco-Zorrilla et al., 2007; Ma et al., 2008). Therefore, to gain further insight into the function of leaf, spike, and grain specific lncRNAs of *T. aestivum*, their co-expression with mRNAs was performed. A total of 68, 29, and 185 lncRNAs showed co-expression with 39, 12, and 75 mRNAs in leaf, spike and grain, respectively (File S5). Most of the co-expressed mRNAs were also found to be stage specific with SI-value more than 0.75. The functional annotation of these mRNAs indicated their role in development and various other metabolic processes. A few highly represented annotations were- receptor like kinases (RLK) such as leucine rich repeat (LRR) (GO: 0005515), wall associated (GO: 0006468, GO: 0005524, GO: 0004672), and cysteine rich repeat (GO:0006468, GO:0005524, GO:0004672) RLKs, photosystem II 10 kDa protein (GO:0009654, GO:0042651, GO:0015979), disease resistant protein RPM1 (GO:0043531) in leaf; pectinesterase/ pectinesterase inhibitor 13 (GO:0005618, GO:0042545, GO:0004857, GO:0030599), Rho guanine nucleotide exchange factor 8 (GO:0005089), and Cytochrome P450 86A2 (GO:0016705, GO:0055114, GO:0020037) in spike; and Serpin Z7 (GO:0005615), rab protein (GO:0006950, GO:0009415), Defensin-like protein 1 (GO:000695), globulin 3A (GO:0045735), Low temperature-induced protein (GO:0016021), WSCI proteinase inhibitor (GO:0009611, GO:0004867) and various predicted proteins in grain. Since these genes play vital role in numerous developmental and other biological processes (Ljungberg et al., 1986; Nordin et al., 1993; Hubert et al., 2003; Okuda et al., 2009; Saito and Ueda, 2009; Gish and Clark, 2011; Francis et al., 2012; Tedeschi et al., 2012; Gu et al., 2016; Gell et al., 2017; Xing et al., 2017); the co-expressed lncRNAs might also be responsible for similar functions.

To gain further insight into the mode of action, we analyzed the chromosomal localization of randomly selected 500 co-expressed lncRNAs and mRNAs. Since the complete sequence of each chromosome of *T. aestivum* is still not known, we could not analyze the location of each transcript. A total of ~57% co-expressed lncRNAs and mRNAs were mapped to the various chromosomes, in which ~47% were located on different set of chromosomes, while ~10% were found on same chromosome. We further analyzed the distance between lncRNAs and mRNAs localized on same chromosome. We found that 7% of them were located at the distance of more than 5 Mb, while 3% were within 5 Mb. The results indicated that the majority of lncRNAs probably involved in regulating the function of genes located on different chromosomes, while a few affect the expression of neighboring genes by *cis*-regulation. Both *cis* and *trans* regulation of gene transcription by lncRNAs has also been reported earlier (Kim and Sung, 2012).

### Modulated expression of lncRNAs under abiotic stress

Earlier studies have shown that various abiotic stress affect the expression of numerous coding and non-coding transcripts, which ultimately resulted into the decreased production (Zhang W. et al., 2014; Asseng et al., 2015; Wang T. et al., 2015; Chung et al., 2016; Shumayla et al., 2016a,b; Taneja et al., 2016). Therefore, we analyzed the expression profile of lncRNAs under heat, drought and salt stress.

**Figure 4:**
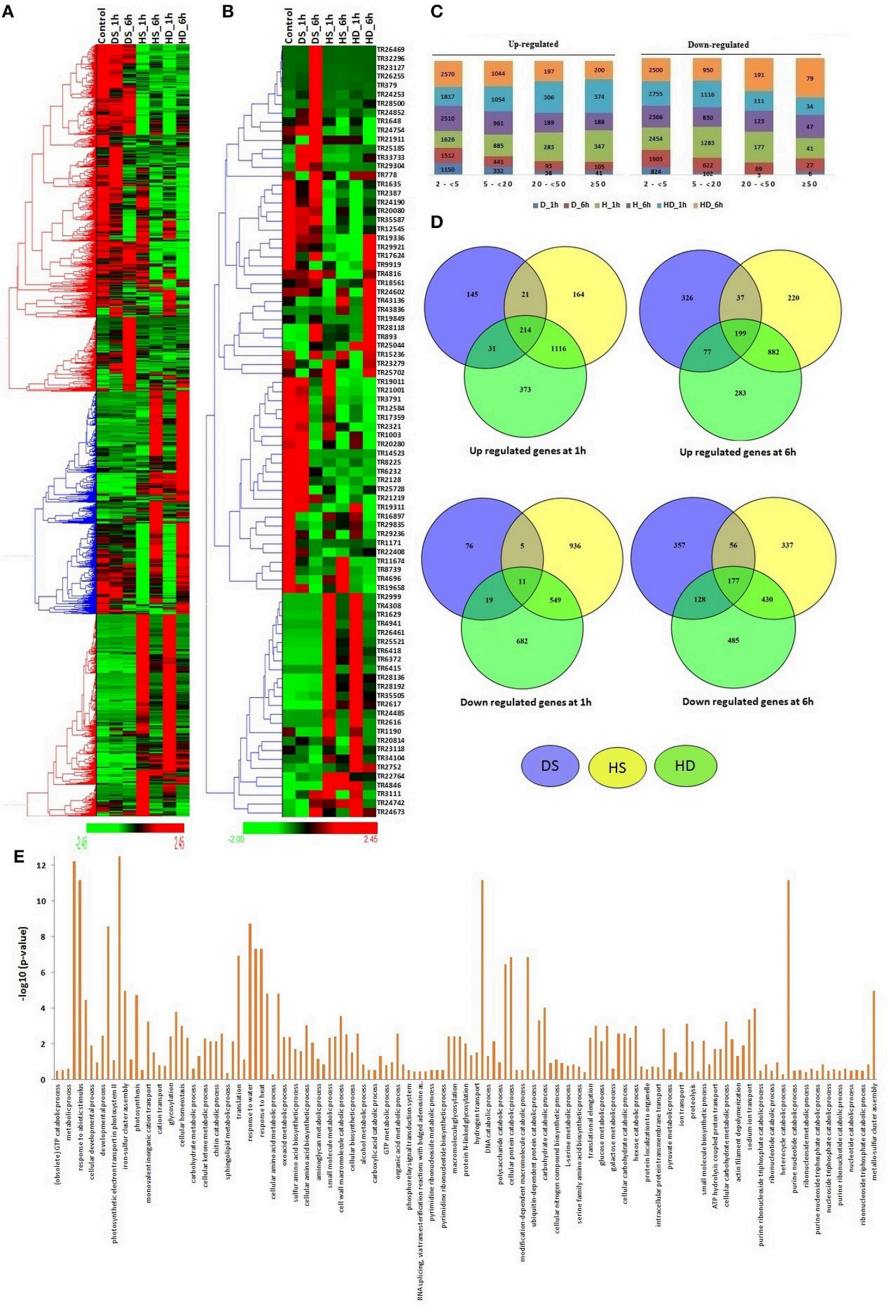
Expression pattern and gene ontology (GO) enrichment analysis of lncRNAs under heat (HS), drought (DS), and their combination (HD) stress. Heat maps show **(A)** expression pattern of each ≥2-fold affected lncRNAs in one or more stress conditions, and **(B)** top affected lncRNAs in various stresses. **(C)** The bar graphs shows the number of up and down regulated lncRNAs in each category of fold expression change (2-<5-fold, 5-<20-fold, 20-<50-fold, and >50-fold). **(D)** Venn diagrams represent the number of ≥5-fold up and down regulated lncRNAs in different condition of HS, DS, and HD. **(E)** The GO enrichment analysis (*P*-value e^−10^) of ≥5-fold affected lncRNAs revealed their role in various biological processes.

The modulated expression of numerous lncRNAs was found during various abiotic stresses (File S6). About 29% lncRNAs were found to be affected (≥2-fold) after heat (HS), drought (DS) and their combination (HD) stress. Likewise, ~26% coding transcripts showed modulated expression during similar conditions (Liu Z. et al., 2015). The results indicated comparable response of both coding and non-coding transcripts during these circumstances. Further a distinct expression pattern of lncRNAs was observed. The majority of lncRNA transcripts showed comparable expression pattern during HS and HD after equal time of treatment, such as the lncRNAs which were up-regulated after HS_1 h and HS_6 h, showed similar trend in HD_1 h and HD_6 h, respectively (Figures 4A,B). Similar correlation in expression pattern under these stress conditions is also reported in case of various protein coding gene families (Liu Z. et al., 2015).

Based on the level of modulation, lncRNAs were classified into four categories- least modulated (2-<5-fold) to the most modulated (>50-folds). A total of 6,258, 2,587, 731, and 532 lncRNAs were up-regulated, whereas 7,617, 3,419, 569, and 199 lncRNAs were down-regulated under one or more stress conditions in various (2 ≤ 5-fold, 5 ≤ 20-fold, 20 ≤ 50-fold, and >50-fold) categories, respectively (Figure 4C). Further analysis was performed using ≥5-fold affected lncRNA transcripts. In-total, 2,064 lncRNAs were up-regulated, while 2,278 lncRNAs were down-regulated after 1 h of DS, HS, and HD treatments (Figure 4D). However, 2,024 and 1,970 lncRNAs were up and down-regulated after 6 h of treatment, respectively. A total of 214 and 199 lncRNAs were commonly up-regulated, while 11 and 177 lncRNAs were commonly down-regulated after 1 and 6 h of treatments, respectively (Figure 4D). Among these, 90 and 7 (TR1171, TR11411, TR11429, TR23050, TR24202, TR2747, and TR32123) lncRNAs were up and down-regulated after both 1 and 6 h of treatments, respectively. The highest up and down-regulated lncRNAs in each treatment were- TR25521 and TR24190 in HS 1 h, TR4846 and TR20814 in HS 6 h, TR22764 and TR25702 in DS 1 h, TR893 and TR34104 in DS 6 h, TR25521 and TR23279 in HD 1 h, and TR893 and TR24754 in HD 6 h, respectively (Figure 4B). The differential expression of lncRNAs after comparable stress treatment are also reported in case of other plant species like Arabidopsis, rice and maize (Liu et al., 2012; Zhang W. et al., 2014; Chung et al., 2016). The results presumed the role of lncRNAs in HS, DS, and HD.

**Figure 5:**
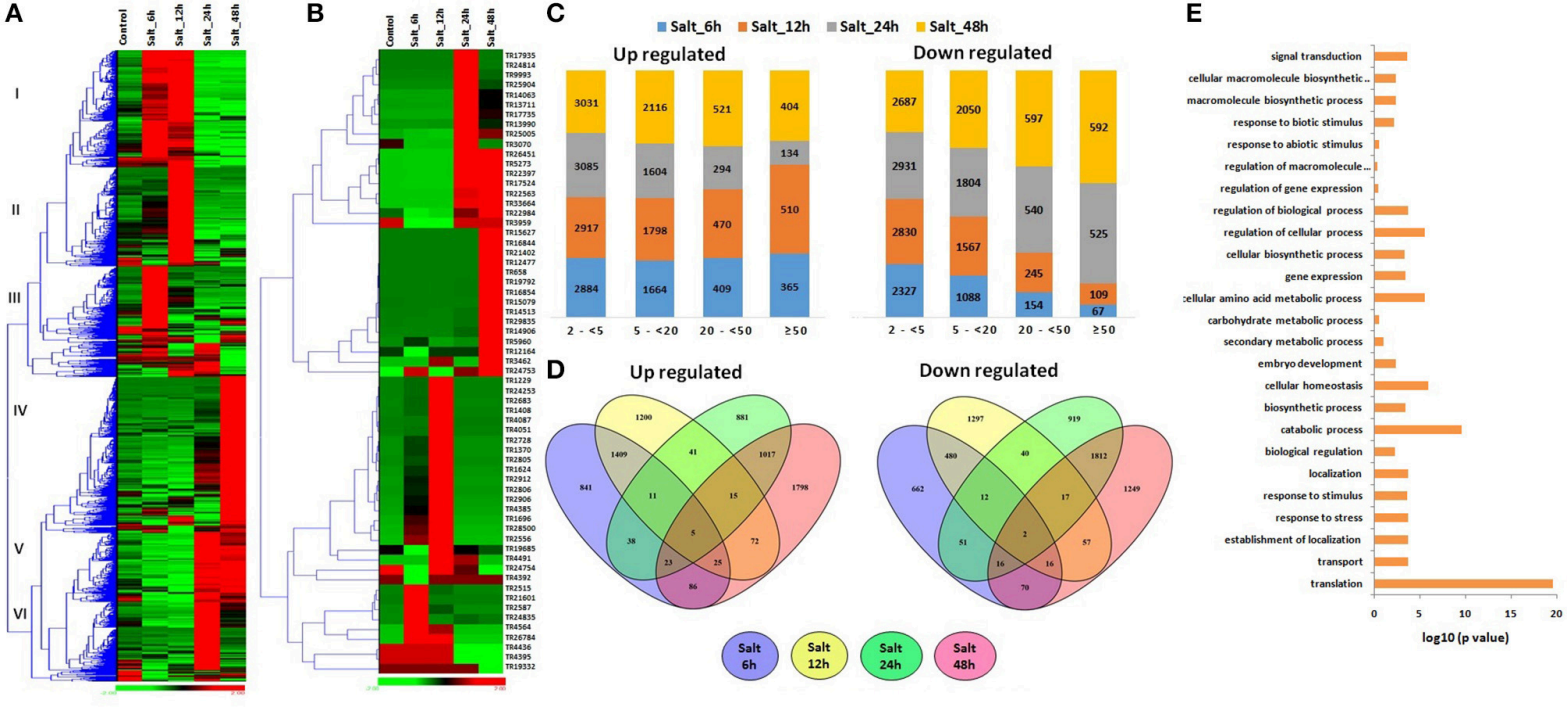
Expression pattern and gene ontology (GO) enrichment analysis of lncRNAs at different time intervals of salt stress. The heat maps show **(A)** the expression pattern of ≥2-fold affected lncRNAs and **(B)** top affected lncRNAs after 6, 12, 24, 48 h of salt stress. **(C)** The bar graphs shows the number of up and down regulated lncRNAs in each category of fold expression change (2-<5-fold, 5-<20-fold, 20-<50-fold, and >50-fold). **(D)** Venn diagrams represent the number of ≥5 fold up and down regulated lncRNAs after 6, 12, 24, 48 h of salt stress. **(E)** The GO enrichment analysis (*P*-value e^−10^) indicated the involvement of salt affected lncRNAs in various biological processes.

To gain further insight into the biological processes related to the HS, DS, and HD affected lncRNAs, the co-expression analysis of ≥5-fold affected lncRNAs with mRNAs was performed, which was further used for GO mapping. A total of 509 affected lncRNAs showed co-expression with respective mRNAs, in which 161 lncRNAs showed co-expression during developmental stages also, but with different set of mRNAs. The GO enrichment analysis of co-expressed genes during HS, DS, and HD indicated their role in various biological and metabolic processes such as response to heat (GO: 0009408), embryo development (GO: 0009790), ion transport (GO: 0006811), protein repair (GO: 0030091), cellular biosynthetic process (GO: 0044249), cellular developmental process (GO: 0048869), cellular homeostasis (GO: 0019725), vitamin biosynthetic process (GO: 0009110) and various other processes (Figure 4E). The results indicated crosstalk between various stress responsive and developmental pathways of plants. The GO enrichment analysis of differentially expressed mRNAs under similar conditions showed comparable pattern, where they were also shown to be involved in response to various abiotic stresses and metabolic processes (Liu Z. et al., 2015). Salt is considered as another major abiotic stress, which affects crop production globally (Zhang Y. et al., 2016). The expression of lncRNAs under salt stress was analyzed at different time intervals (6, 12, 24, and 48 h). A total of 19233 lncRNAs were analyzed for expression profiling owing to their expression value >10 FPKM in at least one treatment (File S7). Numerous genes showed modulated expression in different hours of treatment. An interesting trend was observed after clustering of differentially expressed lncRNAs (Figure 5A). The lncRNAs were clustered into six groups based on their expression pattern. The majority of lncRNAs from group I, II, and III were up-regulated in initial hours (6 and 12 h) of treatment, while down- regulated in later hours (24 and 48 h). Contrarily, most of the group IV, V, and VI lncRNAs were initially down-regulated, however up-regulated in later stages of treatment. Moreover, time specific clustering in expression pattern of lncRNAs was also observed (Figures 5A,B). On the basis of level of differential expression, the lncRNAs were divided into four classes as mentioned above (Figure 5C). Around 35% lncRNAs (15,644/44,698) showed modulated expression by ≥2-fold in one or more treatments, which was comparable to the result reported in case of coding transcripts, where 37% (36,804/99,386) were differentially expressed (Zhang Y. et al., 2016). In-total 9,254, 5,898, 1,512, and 1,101 distinct lncRNAs were up-regulated, whereas 8,417, 5,250, 1,349, and 761 distinct lncRNAs were down-regulated in various categories (2 ≤ 5-fold, 5 ≤ 20- fold, 20 ≤ 50-fold, and >50-fold), respectively (Figure 5C). The ≥5-fold affected lncRNAs transcripts were further analyzed (Figure 5D). Out of 7462 up-regulated lncRNAs, 841, 1,200, 881, and 1,798 distinct lncRNAs were specifically up-regulated after 6, 12, 24, and 48 h of treatment, respectively. Likewise, 662, 1,297, 919, and 1,249 distinct lncRNAs were exclusively down-regulated in above treatments, respectively. Nevertheless, five (TR24835, TR2184, TR3125, TR24194, and TR22177) and two (TR20678 and TR2158) lncRNAs were found as commonly up and down-regulated after each treatment, respectively (Figure 5D). The highly up and down regulated lncRNAs in each condition of salt stress were- TR2912 and TR17524 after 6 h, TR2728 and TR25005 after 12 h, TR17935 and TR2912 after 24 h, and TR15627 and TR1229 after 48 h of treatment, respectively (Figure 5B, File S7). The modulated expression of lncRNAs after salt stress is also reported in other plant species. In-total, 2,477 lncRNAs were up regulated during salt stress in leaves of *M. truncatula* (Wang T. et al., 2015). The over expression of *npc536* lncRNA in Arabidopsis increased root growth under salt stress (Amor et al., 2009). The results revealed involvement of lncRNAs in salt stress response.

To further characterize the role of differentially expressed lncRNAs, co-expression and GO enrichment analysis was performed as described above. *In-total* 1,853 lncRNAs showed co-expression with respective mRNAs during salt stress, in which 381 and 32 lncRNAs also showed co-expression during developmental stages and heat-drought stress with different set of mRNAs, respectively. Further, 14 lncRNAs co-expressed during each condition. The results indicated temporal and spatial changes in the expression profiles of lncRNAs. These differentially expressed lncRNAs were predicted to be associated with diverse functions like response to stress (GO: 0006950), response to abiotic (GO: 0009628) and biotic (GO: 0009607) stimulus, transport (GO: 0006810), embryo development (GO: 0009790), gene expression (GO: 0010467), biological regulation (GO: 0650007), and various biosynthetic processes (GO: 0009058, GO: 0009059) (Figure 5E).

The chromosomal mapping of co-expressed lncRNAs and mRNAs during heat-drought and salt stress showed similar trend as observed in case of developmental stages. *In-total* ~53 and ~60% co-expressed lncRNAs and mRNAs were located on various chromosomes during heat-drought and salt stress, in which ~43 and ~49% were mapped on different chromosomes, respectively. The results indicated both intra and inter chromosomal regulation of gene expression by lncRNAs.

### Transcription factors showing co-expression with lncRNAs

**Figure 6:**
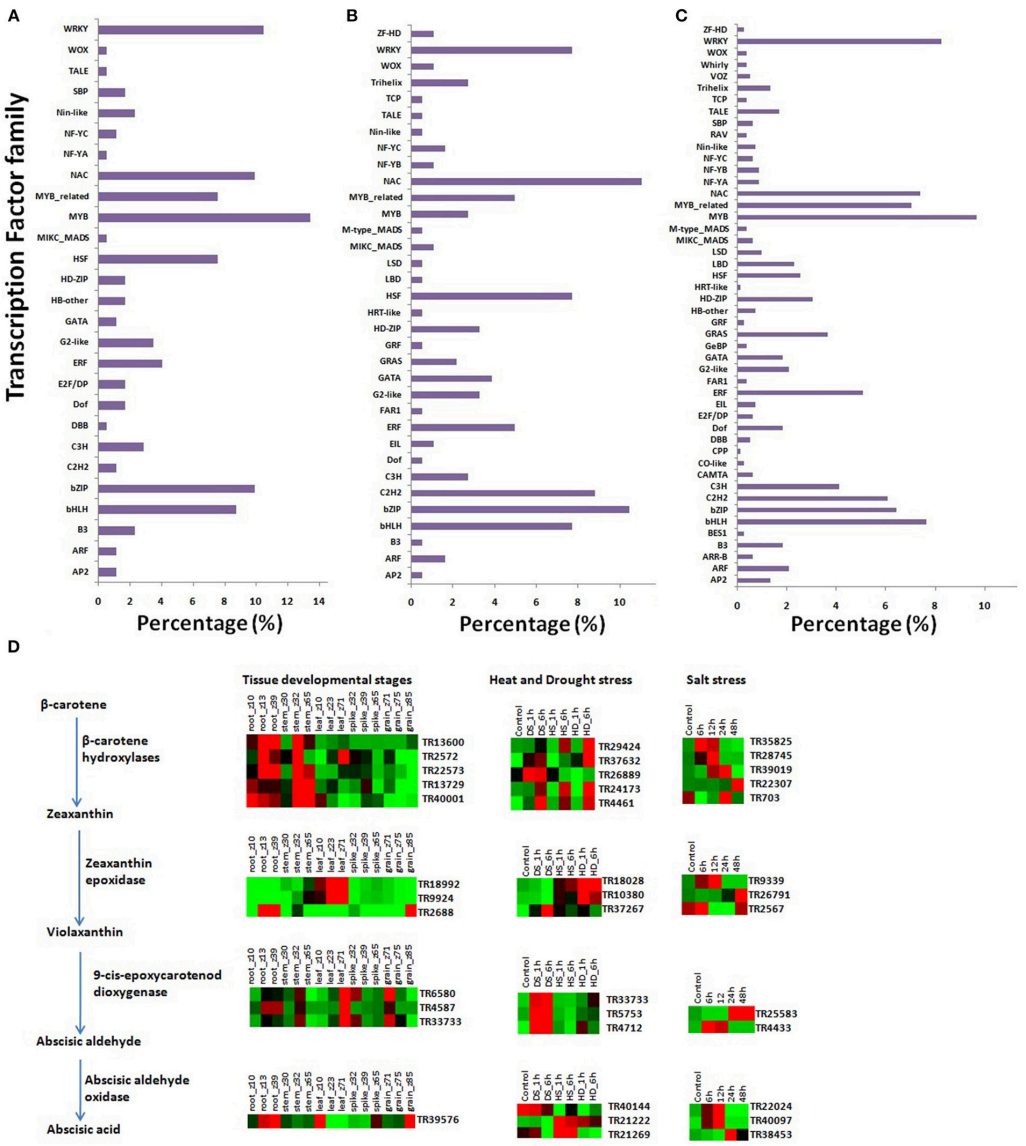
Transcription factor families co-expressed with lncRNAs, and expression analysis of lncRNAs involved in Abscisic acid biosynthesis. Figure shows occurrence of various TF families showing co-expression with lncRNA during development **(A)**, heat-drought **(B)**, and salt **(C)** stress. **(D)** Figure shows enzymes involved in various steps of ABA biosynthesis, and relative expression profile of co-expressed lncRNAs during development and stress conditions.

Transcriptions factors (TFs) are a group of important regulatory proteins which are involved in regulation of numerous developmental and stress related pathways in plants (Saibo et al., 2009; Lindemose et al., 2013; Wang et al., 2016). The sequence information of TFs of *T. aestivum* was obtained from Plant Transcription Factor Database (http://planttfdb.cbi.pku.edu.cn/, Jin et al., 2017) and used for co-expression analysis with lncRNAs. A total of 172 TFs belonging to the 27 TF families were found co-expressed with lncRNAs during various developmental stages (Figure 6A). The TFs belonging to WRKY, NAC, MYB, HSF, Bzip, and bHLH families were found to be highly enriched during developmental stages. Moreover, TFs related SBP, Nin-like, G2-like, ERF, C3H, B3, and others were also found co-expressed with certain lncRNAs. These co-expressed TFs were reported earlier to be involved in numerous developmental processes in plants (Shikata et al., 2009; Lindemose et al., 2013; Makkena and Lamb, 2013; Guo et al., 2016).

In case of abiotic stresses like heat-drought (HS, DS, and HD) and salt, only ≥5-fold affected lncRNAs were used for co-expression analysis. *In-total*, 181 and 828 TFs related to 34 and 48 different families showed co-expression with lncRNAs during these stresses, respectively (Figures 6B,C). Though, the number of TFs and TF families co-expressed during salt stress was higher than the heat-drought, but the occurrence pattern was comparable. The TFs related to the WRKY, NAC, MYB_related, HSF, ERF, C2H2, bZIP, and bHLH families were highly represented during heat-drought stress. Similarly, WRKY, NAC, MYB_related, MYB, ERF, C3H, C2H2, bZIP, and bHLH TF families were more enriched in salt stress (Figures 6B,C). These TFs are known to be involved in stress response in plants (Saibo et al., 2009; Lindemose et al., 2013; Makkena and Lamb, 2013; Guo et al., 2016; Wang et al., 2016). Further analysis indicated differential enrichment of various Auxin and ABA related TFs like ARF, bZIP, bHLH, ERF, HD-Zip MYB, and others during development and abiotic stresses. For instance, ARF, bZIP, ERF TFs were found enriched during each abiotic stress than development, while MYBs were reduced during heat-drought stress. These hormone responsive TFs are involved in diverse functions in plants (Chapmen and Estelle, 2009; Chew et al., 2013; Chen et al., 2017). The co-expression of lncRNAs with these TFs further indicated their putative role in various regulatory processes.

### Role of lncRNAs in abscisic acid biosynthesis and signaling

Since abscisic acid (ABA) plays central role in numerous abiotic stresses (Saibo et al., 2009), we analyzed the co-expression of four important enzymes (BCH, ZEP, NCED, AAO) encoding mRNAs with lncRNAs. The BLAST search of BCH, ZEP, NCED, AAO sequences from Arabidopsis (Finkelstein, 2013) against *T. aestivum* gene model sequences identified a total of 5 (Traes_4AL_C4C082F05.1, Traes_4DL_5BBB45AF3.1, Traes_4BL_EB421721D.1, Traes_7DL_6D8026529.1, and Traes_7BL_FF177DF09.1), 3 (Traes_2AL_5FF7D6940.1, Traes_2BL_047344CB6.1, and Traes_2DL_7A9E10914.1), 3 (Traes_5BL_4BED1CA17.1, Traes_5BS_B626C522B.1, and Traes_5DS_E58EBABFD.1), and 3 (Traes_7AL_3363AD1E0.1, Traes_7DL_9A293EA4D.1, and Traes_7DL_E5EECE349.1) orthologous mRNA sequences for each enzyme, respectively. The identified *T. aestivum* mRNAs showed co-expression with 12, 14, and 13 different lncRNAs during development, and heat-drought and salt stresses, respectively (Figure 6D). The co-expression of these lncRNAs with ABA biosynthesis related mRNAs, differential expression in various developmental stages, and modulated expression during abiotic stresses indicated their putative role in ABA biosynthesis. However, their precise mode of action is not known.

The ABA is responsible for the signaling of numerous abiotic stress conditions in plants by activating various TFs (Saibo et al., 2009; Lindemose et al., 2013). The co-expression of certain lncRNAs with ABA dependent TFs such as MYB, WRKY, bZIP, bHLH, C2H2, ERF, and others (Figures 6B,C) during heat-drought and salt stresses signifies their role in ABA signaling. Moreover, certain lncRNAs showed co-expression with ABA independent TFs like NAC and ZF-HD, which might be responsible for ABA independent signaling.

### Analysis of lncRNAs function as precursor and target mimic of miRNAs

**Figure 7:**
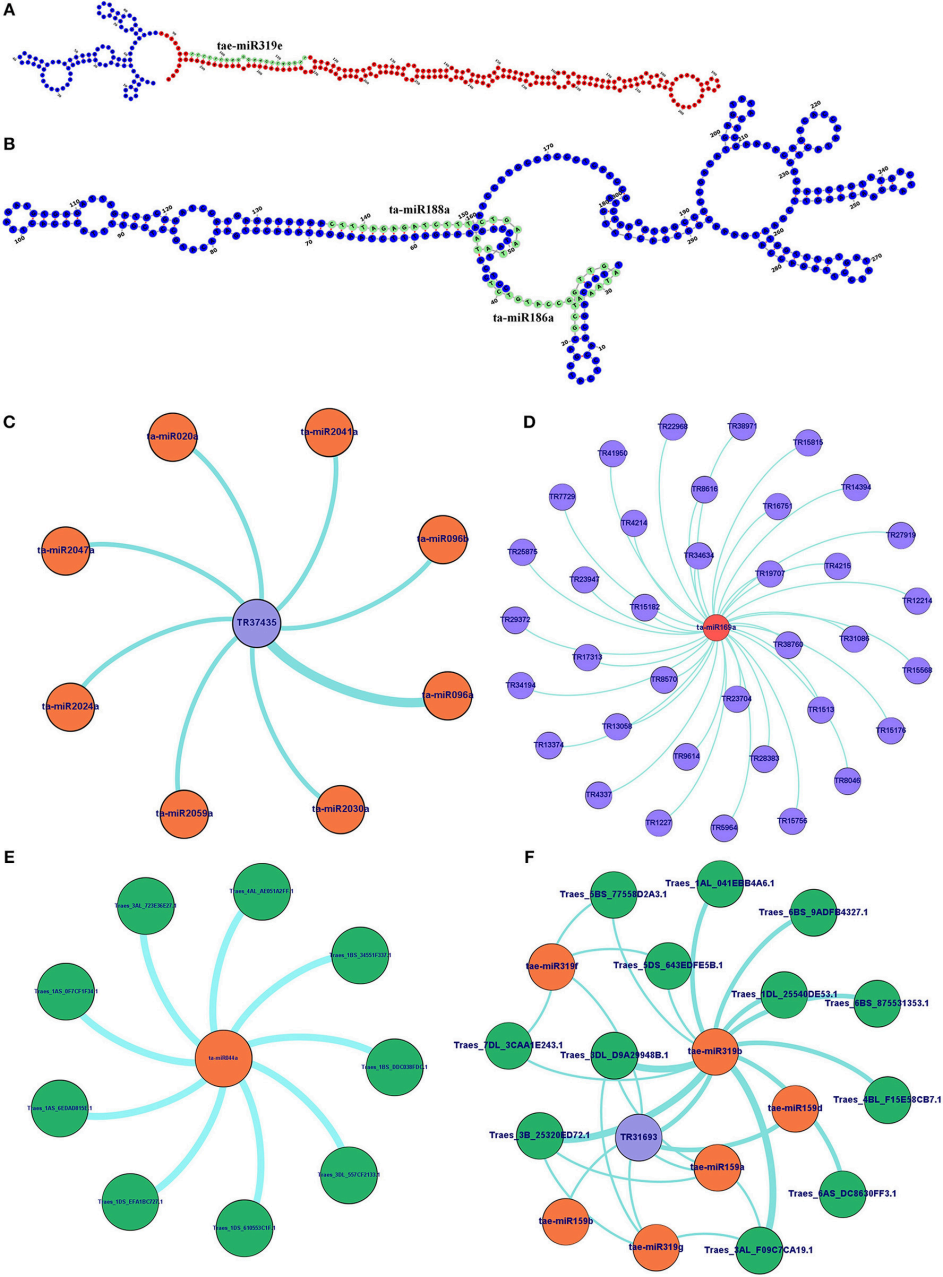
Secondary structure and interaction analysis of lncRNAs with miRNAs and mRNAs. **(A)** Figure shows secondary structure of lncRNA (TR31693), which act as putative precursor of miRNA (taemiR319e). The precursor and mature miRNA regions are marked with red and green colors, respectively. **(B)** Secondary structure of lncRNA (TR20073) acts as target mimic for two miRNAs (ta-mir188a and ta-mir186b) shown in green color. Figures show **(C)** interaction of an lncRNA with multiple miRNAs, and interaction of miRNA with multiple lncRNAs **(D)** and mRNAs **(E)**. **(F)** An interaction network shows association between lncRNAs, miRNAs and mRNAs. The complete network is shown in Figure S3. The lncRNA, miRNAs, and mRNAs are represented in blue, orange, and green circles, respectively.

**Figure 8:**
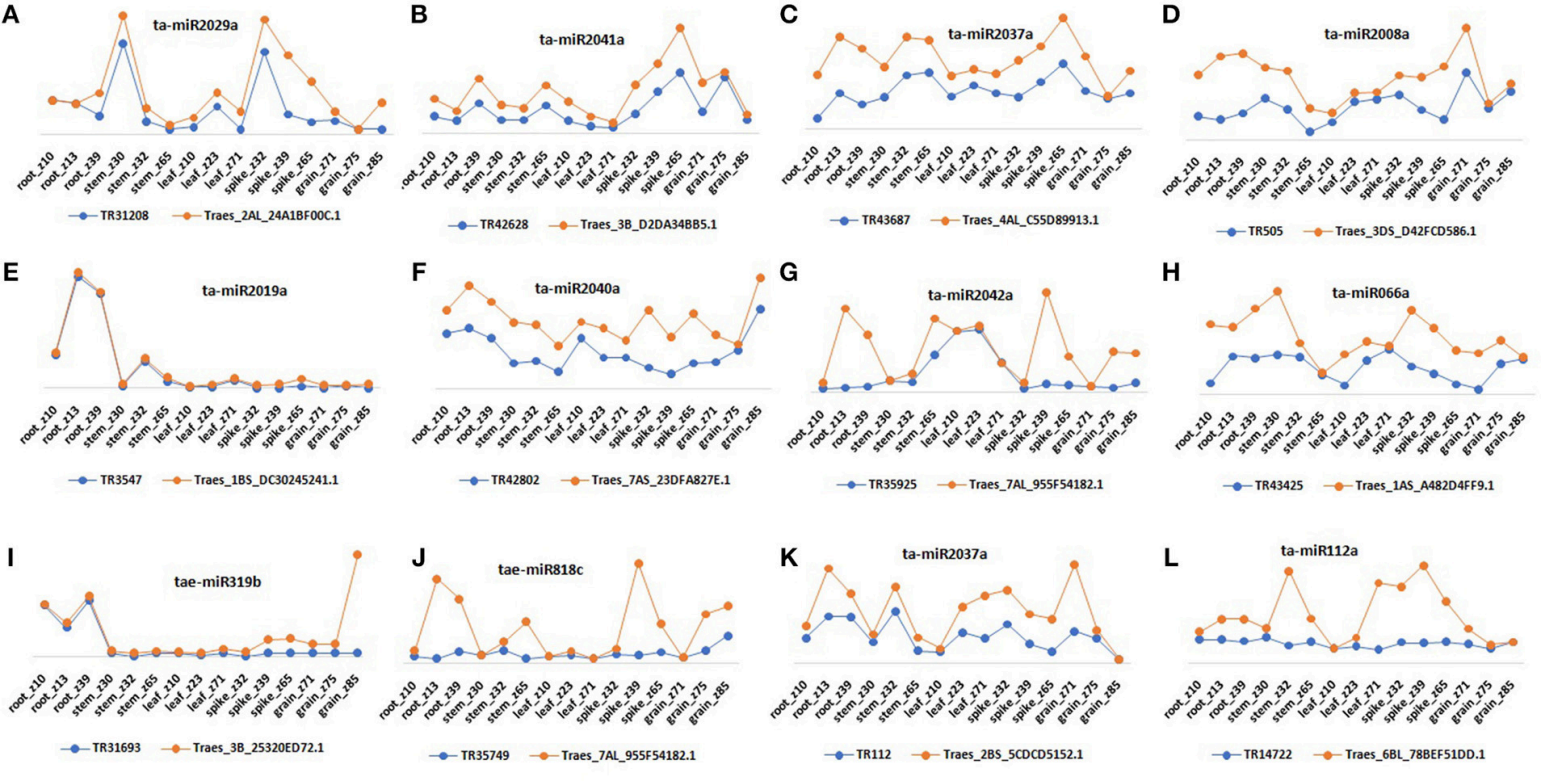
Relative expression profiling of selected lncRNA and mRNA pairs that act as target and potential mimics for common miRNA. Figure shows expression pattern of miRNAs **(A)** ta-miR2029a, **(B)** ta-miR2041a, **(C)** ta-miR2037a, **(D)** ta-miR2008a, **(E)** ta-miR2019a, **(F)** ta-miR2040a, **(G)** ta-miR2042a, **(H)** ta-miR066a, **(I)** tae-miR319b, **(J)** tae-miR818c, **(K)** ta-miR2037a, and **(L)** ta-miR112a interacting mRNAs and lncRNAs in various tissue developmental stages.

Since the lncRNAs also function through miRNAs for transcriptional, post-transcriptional and epigenetic gene regulation through diverse molecular mechanisms (Rinn and Chang, 2012). We explored the lncRNAs acting as precursor and target mimic of known miRNAs in *T. aestivum*. A total of 19 lncRNAs were predicted as precursor of 28 miRNAs (Figure 7A, Figure S2). Thirteen lncRNAs were predicted as precursor of single miRNA, while 4 (TR23494, TR24856, TR115, and TR29119), 1 (TR34536), and 1 (TR5782) were acted as precursor of 2, 3, and 4 miRNAs, respectively. Further, lncRNAs regulate the gene expression and ultimately the numerous biological processes by acting as target mimic or decoy of miRNA (Johnsson et al., 2013; Paraskevopoulou et al., 2013; Gupta, 2014; Fan et al., 2015). Therefore, the interaction analysis among lncRNAs, miRNAs and mRNAs was carried out. A total of 1,047 lncRNAs showed interaction with 222 miRNAs of *T. aestivum* (File S8), which were further interacted with 209 distinct mRNA transcripts involved in various functions (Figures S3, S4, Files S8, S9). Multiple sets of interactions were detected such as single lncRNA (TR37435) interacted with multiple miRNAs (Figures 7B,C), single miRNA interacted with several lncRNAs (Figure 7D) and mRNAs (Figure 7E), and a network of interaction among lncRNAs, miRNAs and mRNAs (Figure 7F, Figure S3). Out of 1047 miRNA interacting lncRNAs; 149, 44, and 20 lncRNAs were found differentially expressed during development, salt stress and heat-drought stress, respectively. Similar interaction pattern has been reported in other plant species like maize and chickpea, where 78 and 236 miRNAs interacted with 117 and 144 lincRNAs, respectively (Fan et al., 2015; Khemka et al., 2016). The GO enrichment analysis of lncRNAs and miRNAs interacting mRNAs of *T. aestivum* indicated that majority of them are involved in numerous regulatory processes such as regulation of gene expression (GO:0010468), metabolic (GO:0031323, GO:0019222), cellular (GO:0050794) and biological (GO:0065007, GO:0050789) processes and various biosynthetic processes (GO:0032774, GO:0010556, GO:0009889; Figure S4). Functional characterization of a few miRNA interacting lncRNAs has been performed in certain plants. For instance, sequestration of miR-399 by target mimic *IPS1* lncRNA resulted in increase in the accumulation of target *PHO2* mRNA in Arabidopsis (Franco-Zorrilla et al., 2007). Further, an increase in *IPS1* concentration was positively correlated with *PHO2* accumulation. However, detailed functional validation of lncRNAs is negligible in comparison to the coding genes. The expression profiling of selected miRNAs targets (mRNA) and predicted potential mimics (lncRNA) displayed similar correlation in most of the tissue developmental stages in *T. aestivum* (Figures 8A–L). For instance, ta-miR2029a interacting mRNA “Traes_2AL_24A1BF00C.1” and lncRNA “TR31208” exhibited positive correlation in expression trend in each tissue developmental stages (Figure 8A). Moreover, a negative correlation or no correlation could also be observed in certain cases, which might be either due to complex interaction network between lncRNAs, miRNAs and mRNAs or due to some unknown regions.

Further, out of 209 lncRNAs interacting mRNAs, 19 encodes for TFs such as SBP (7), NAC (6), MYB (2), MIKC_MADS (2), bHLH (1), and ERF (1), which are reported to be involved in various developmental and stress related pathways (Saibo et al., 2009; Shikata et al., 2009; Wang et al., 2016). The association of lncRNAs with miRNAs and mRNAs suggested their vital functions in plants, but the precise role of individual lncRNAs needs to be validated in future studies.

## Conclusions

The lncRNAs play vital functions in growth and development, and stress response in numerous organisms. They are characterized in detail in animal system, but rarely studied in plants. Advances in various sequencing technology and methods of computational analysis enabled characterization of lncRNAs in a few plant species. However, they were partially analyzed in *T. aestivum*, which is an important food crop. It is probably due to the lack of genomic information and unavailability of a proper pipeline for the identification of lncRNAs. In the last few years, a consensus has been developed about the various criteria for the identification of lncRNAs, which enable identification of these in unconventional plant species. Further, unlike the coding RNAs, lncRNAs cannot be identified directly from genome sequence due to the lack of defining features such as promoter elements and transcript properties, required by the prediction algorithms. They can be only identified from RNA seq data. Furthermore, the temporal, spatial, inducible and various other specific expression patterns of lncRNAs reduced the probability of comprehensive identification using the data generated from a specific developmental stage or treatment. Therefore, most of the studies in plants are performed at limited scale. Herein, we performed identification of lncRNAs in *T. aestivum* using the data generated in diverse conditions including developmental stages and various abiotic stress treatments. Moreover, it is still at limited scale in many aspects. A total of 44,698 lncRNAs were identified in *T. aestivum* genome after the analysis of 52 RNA seq data, which were distributed throughout the various sub-genomes and chromosomes. The lncRNAs were found to be rich in AU content as compared to mRNAs. Also, 7,743 lncRNAs were functionally annotated, which predicted their role in photosynthesis, response to various biotic and abiotic stimuli, and various other processes. Similar to the mRNA, the lncRNAs also showed differential as well as tissue developmental stage specific expression, which indicated their role in numerous developmental processes. About 2% lncRNAs were found to be developmental stage specific. Heat, drought and their combination stress modulated ≥2-fold expression of ~29% lncRNAs, however salt stress affected ~37% lncRNAs, which was comparable to the results reported in case of mRNAs. The gene ontology mapping enlightened the probable function of these differentially expressed lncRNAs, however the actual role needs to be established in future studies. The chromosomal localization analyses of co-expressed lncRNAs and mRNAs indicated both *cis* and *trans* regulation of gene expression by lncRNA. Further, the functional characterization of lncRNAs is a challenging task especially in case of non-model plant species, because it can only be performed in species of their origin, due to the high nucleotide sequence divergence between different species. The co-expression of a few lncRNAs with various TFs involved in numerous developmental and stress pathways indicated that they might be associated with similar function. Certain lncRNAs also showed co-expression with enzymes involved in ABA biosynthesis, which is an important hormone involved in stress management in plants. Further, some lncRNAs were predicted as precursor, and as target mimics for certain miRNAs of *T. aestivum*. The lncRNAs interacting miRNAs were further interacted with mRNA, which indicated their putative modus-operandi. A few lncRNAs could interact with multiple miRNAs and vice versa. The results open an opportunity for the designing of synthetic lncRNAs, which may act as miRNA sponge or miRNA scavenger to establish biological functions of a network of genes at a time. It can also be utilized for various crop improvement programs. Numerous functions predicted for lncRNAs of *T. aestivum* needs to be individually established in future studies. This study paves the way for further understanding of their role in regulatory mechanism of various developmental processes and stress management in plants.

## Author contributions

SKU conceived the idea and designed the experiments. SS, S, MT, and ST performed the experiments. SKU, SS, S, MT, ST, and KS analyzed the data. SKU, S, MT, and ST wrote the manuscript, and SKU and KS finalized the manuscript.

### Conflict of interest statement

The authors declare that the research was conducted in the absence of any commercial or financial relationships that could be construed as a potential conflict of interest.

## Supplementary material

Figure S1 Relative expression profile of mRNAs in various tissue developmental stages. **(A)** Heat map shows the relative expression pattern of mRNAs having expression value >10 FPKM in at least one stage in three developmental stages of five tissues (root, leaf, stem, spike and grain). The developmental stages are shown in Zadoks scale. **(B)** Distribution of mRNAs in various categories on the basis of expression level in different developmental stages. **(C)** Heat map shows relative expression profile of top expressing mRNAs from various developmental stages. **(D)** Pie chart shows percentage of mRNAs having specific expression in various tissues. Spike shows highest proportion of specifically expressed mRNAs, which is followed by grain.

Figure S2 Secondary structure of lncRNAs predicted as precursor of various miRNAs. The precursor and mature miRNA regions are marked with red and green colors, respectively.

Figure S3 Complete interaction network of lncRNAs with miRNAs and mRNAs. The lncRNA, miRNAs and mRNAs are represented in blue, orange and green circles, respectively.

Figure S4 GO enrichment analysis of mRNA having miRNA mediated interactions with lncRNAs.

File S1 List of RNA seq data used in analysis.

File S2 List of various characteristic features of identified lncRNAs of *Triticum aestivum*.

File S3 Annotation of lncRNAs of *T. aestivum* by co-expression and gene ontology (GO) mapping.

File S4 Tissue specificity index (TSI) of lncRNAs in various tissue developmental stages. Transcripts having expression value <10 FPKM in each stage were discarded. The TSI-value >0.75 is highlighted.

File S5 List of co-expressed mRNA of leaf, spike and grain specific lncRNAs, TSI-value in respective tissue and functional annotation.

File S6 Fold expression change in lncRNAs of *T. aestivum* during drought (DS), heat (HS) and their combination (HD) stress. Various folds categories are highlighted in different colors. The up and down regulated categories are shown with upward and downward arrows, respectively.

File S7 Fold expression change in lncRNAs of *T. aestivum* during salt stress. Various folds categories are highlighted in different colors. The up and down regulated categories are shown with upward and downward arrows, respectively.

File S8 Prediction of lncRNA as target mimic of known miRNA using psRNATarget (http://plantgrn.noble.org/psRNATarget/) server (Dai and Zhao, 2011). The known miRNA of *T. aestivum* was obtained from Sun et al. (2014).

File S9 Prediction of known miRNA target in lncRNA and mRNA sequences of *T. aestivum*. The known miRNA of *T. aestivum* was obtained from Sun et al. (2014).
