# Supplementary figures and images for: Survey of High Throughput RNA-Seq Data Reveals Potential Roles for lncRNAs during Development and Stress Response in Bread Wheat
Source: Front Plant Sci. 2017 Jun 9;8:1019. doi: 10.3389/fpls.2017.01019 (PMC5465302; doi:10.3389/fpls.2017.01019)

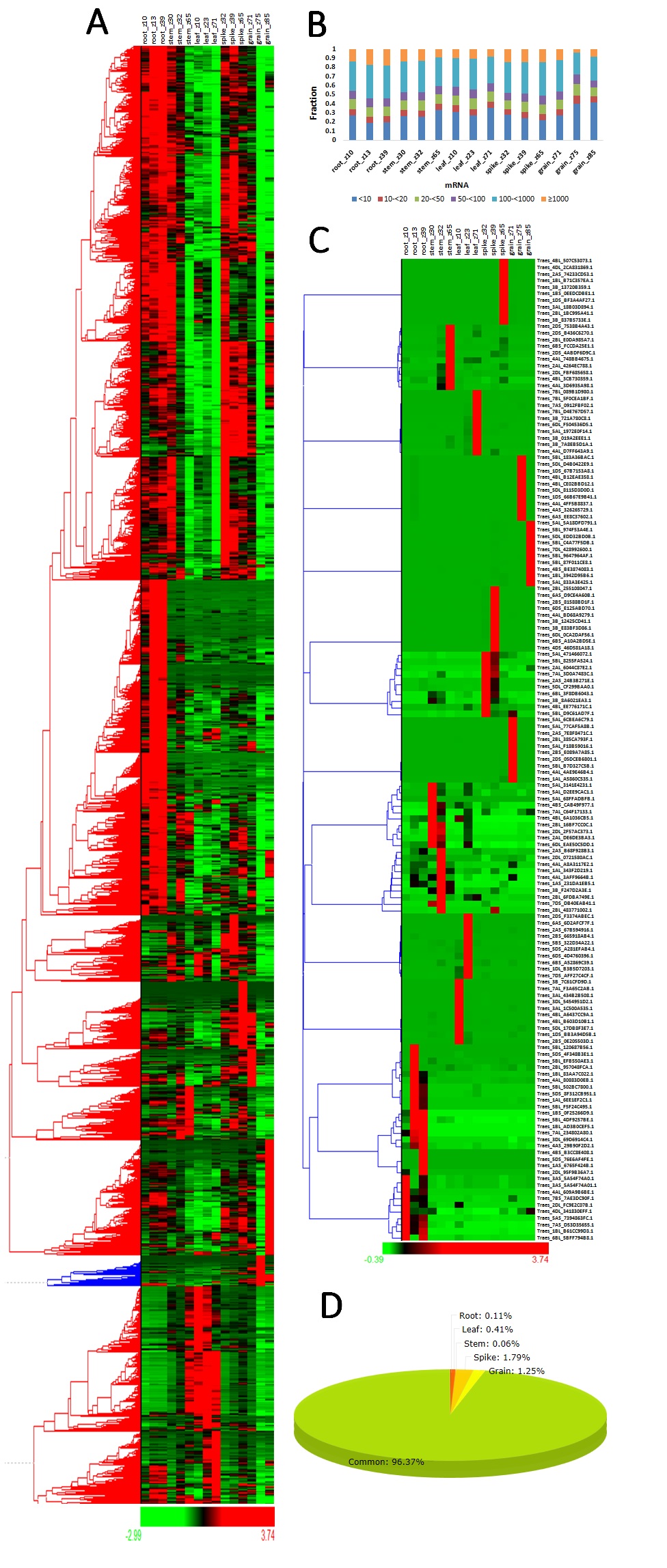

Supplement: Figure S1 — Relative expression profile of mRNAs in various tissue developmental stages. (A) Heat map shows the relative expression pattern of mRNAs having expression value >10 FPKM in at least one stage in three developmental stages of five tissues (root, leaf, stem, spike and grain). The developmental stages are shown in Zadoks scale. (B) Distribution of mRNAs in various categories on the basis of expression level in different developmental stages. (C) Heat map shows relative expression profile of top expressing mRNAs from various developmental stages. (D) Pie chart shows percentage of mRNAs having specific expression in various tissues. Spike shows highest proportion of specifically expressed mRNAs, which is followed by grain. [file Image1.JPEG]

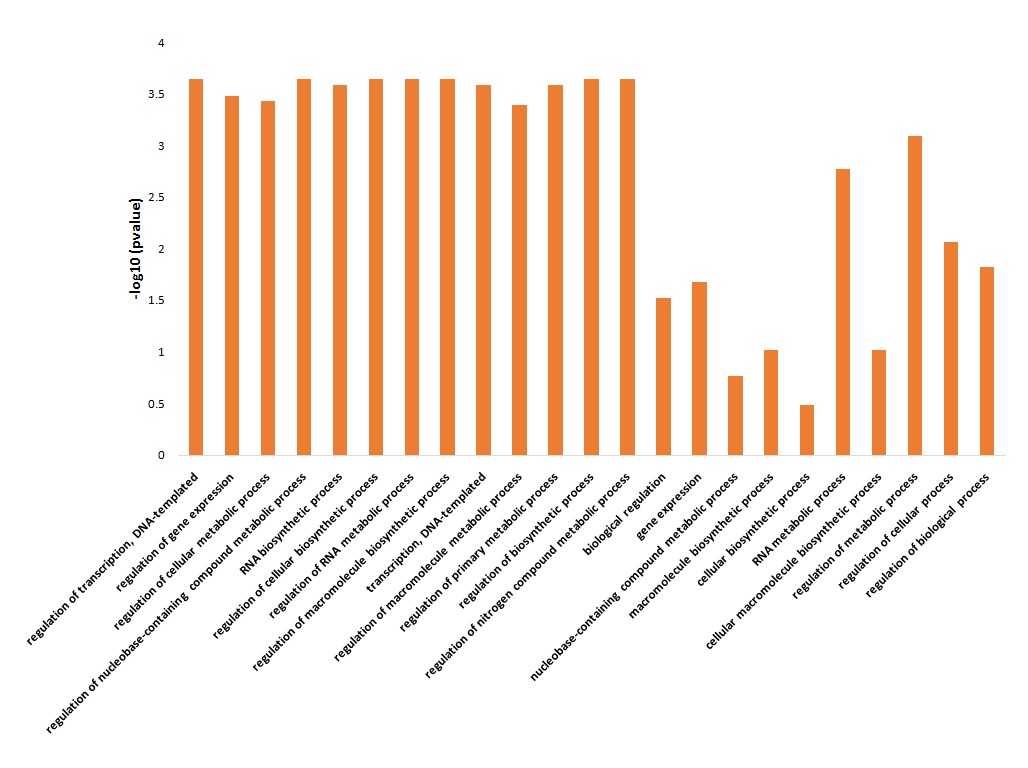

Supplement: Figure S4 — GO enrichment analysis of mRNA having miRNA mediated interactions with lncRNAs. [file Image4.JPEG]
