# Supplementary material for: Survey of High Throughput RNA-Seq Data Reveals Potential Roles for lncRNAs during Development and Stress Response in Bread Wheat
Source: Front Plant Sci. 2017 Jun 9;8:1019. doi: 10.3389/fpls.2017.01019 (PMC5465302; doi:10.3389/fpls.2017.01019)
Supplement: Figure S2 — Secondary structure of lncRNAs predicted as precursor of various miRNAs. The precursor and mature miRNA regions are marked with red and green colors, respectively. [file Image2.PDF]

Figure S2 Secondary structure of lncRNAs predicted as precursor of various miRNAs. The precursor and mature miRNA regions are marked with red and green colours, respectively.

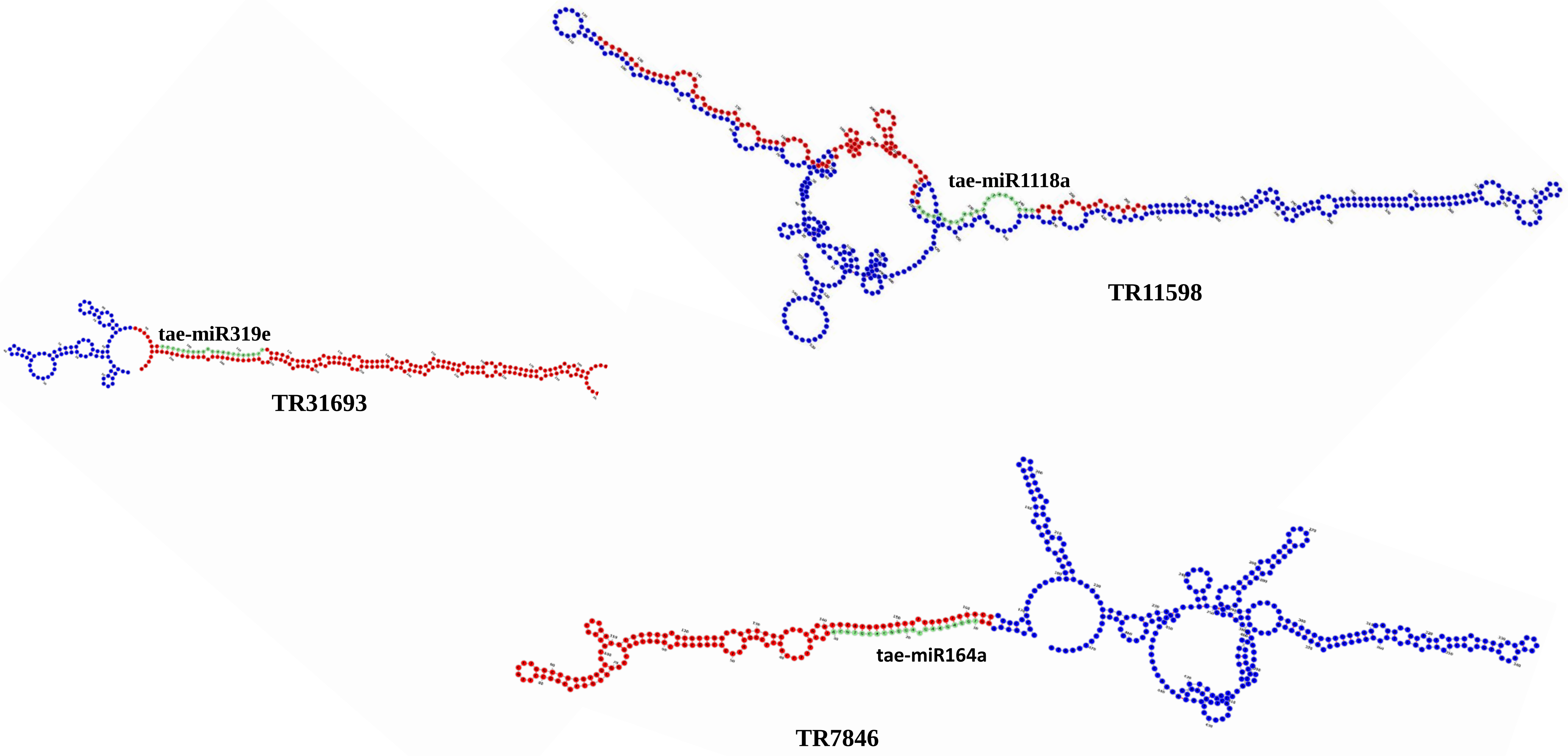

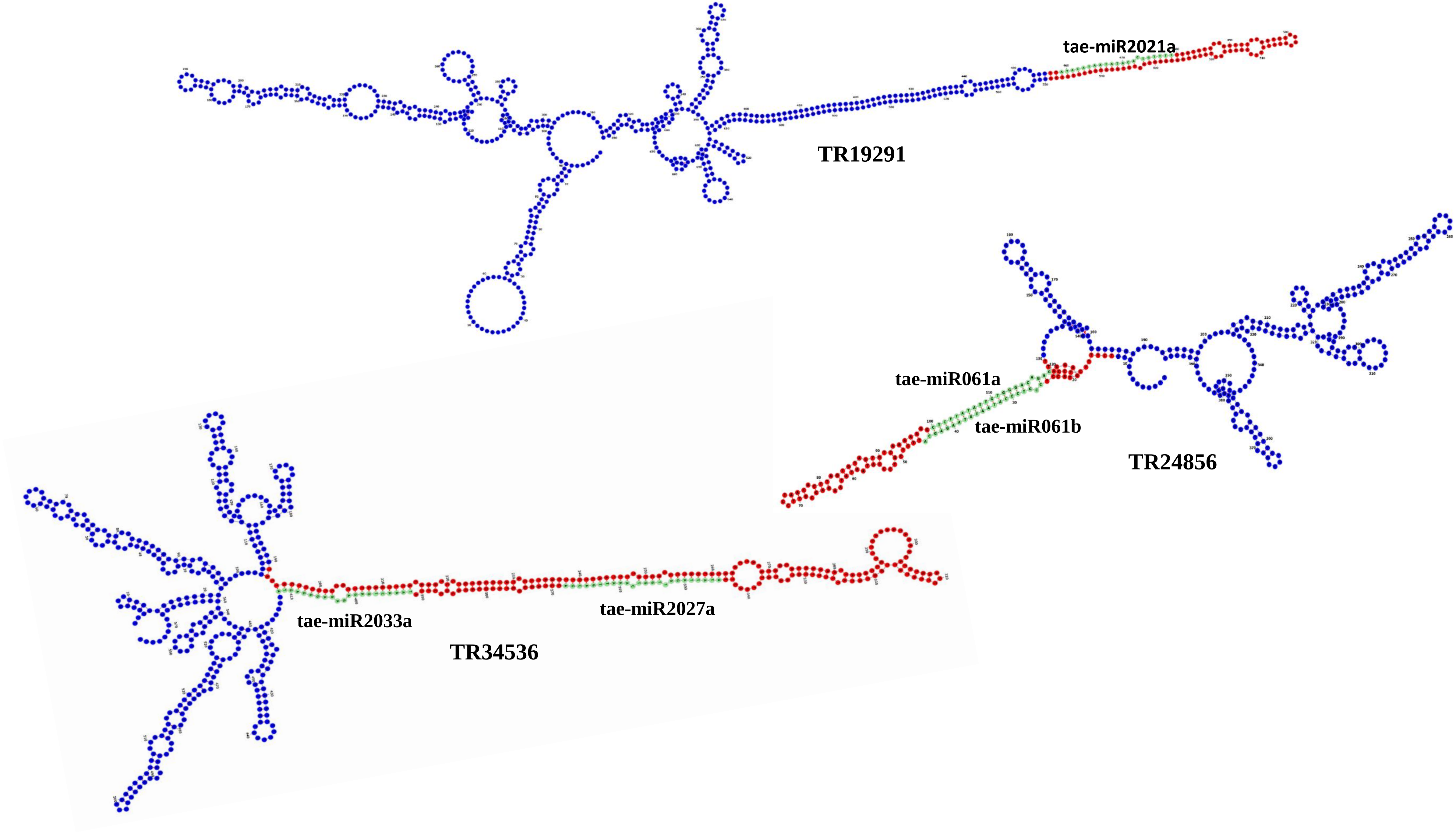
tae-miR2021a
TR19291
tae-miR061a
tae-miR061b
TR24856
tae-miR2033a
tae-miR2027a
TR34536

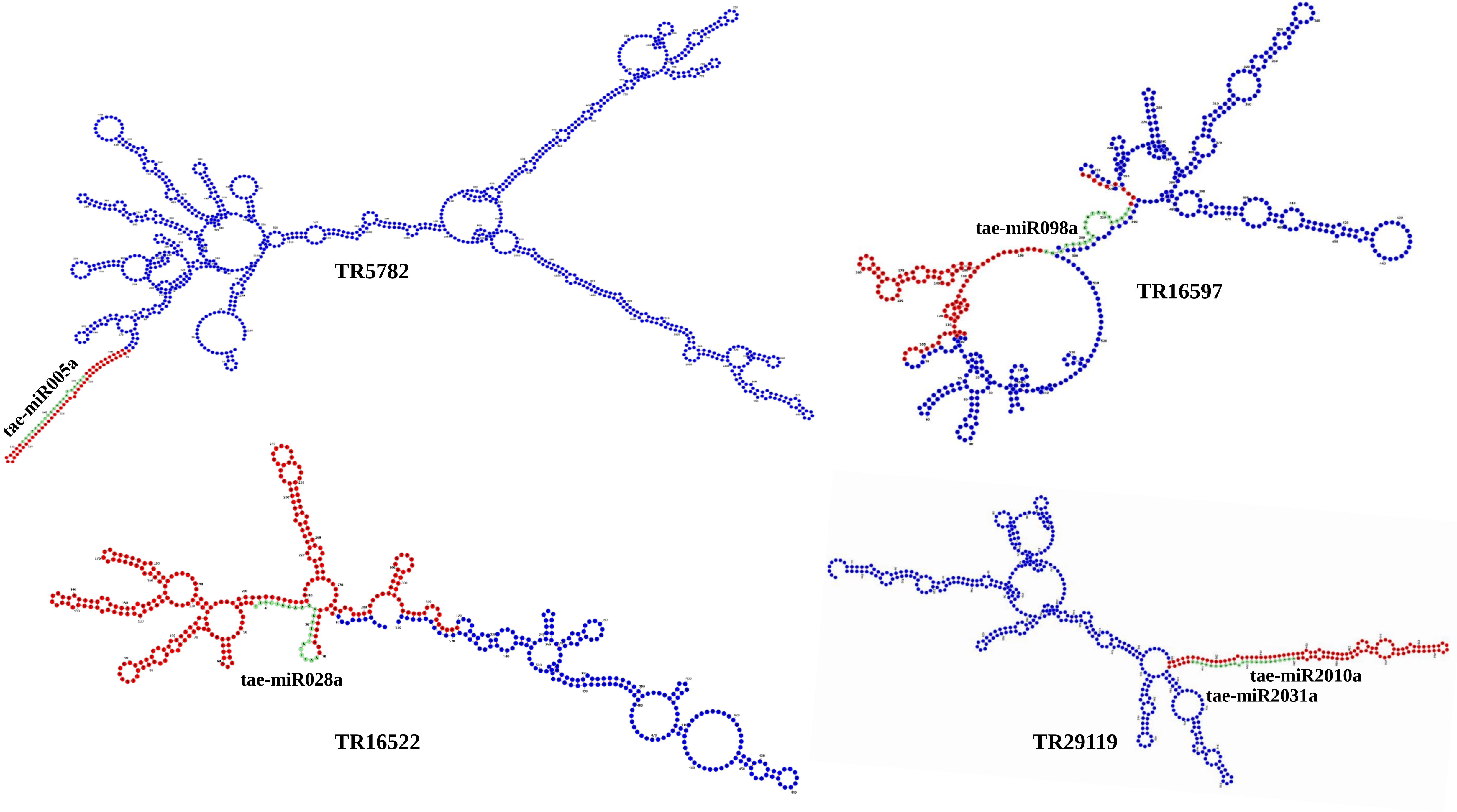

TR5782
tae-miR005a
tae-miR098a
TR16597
tae-miR028a
TR16522
tae-miR2010a
tae-miR2031a
TR29119

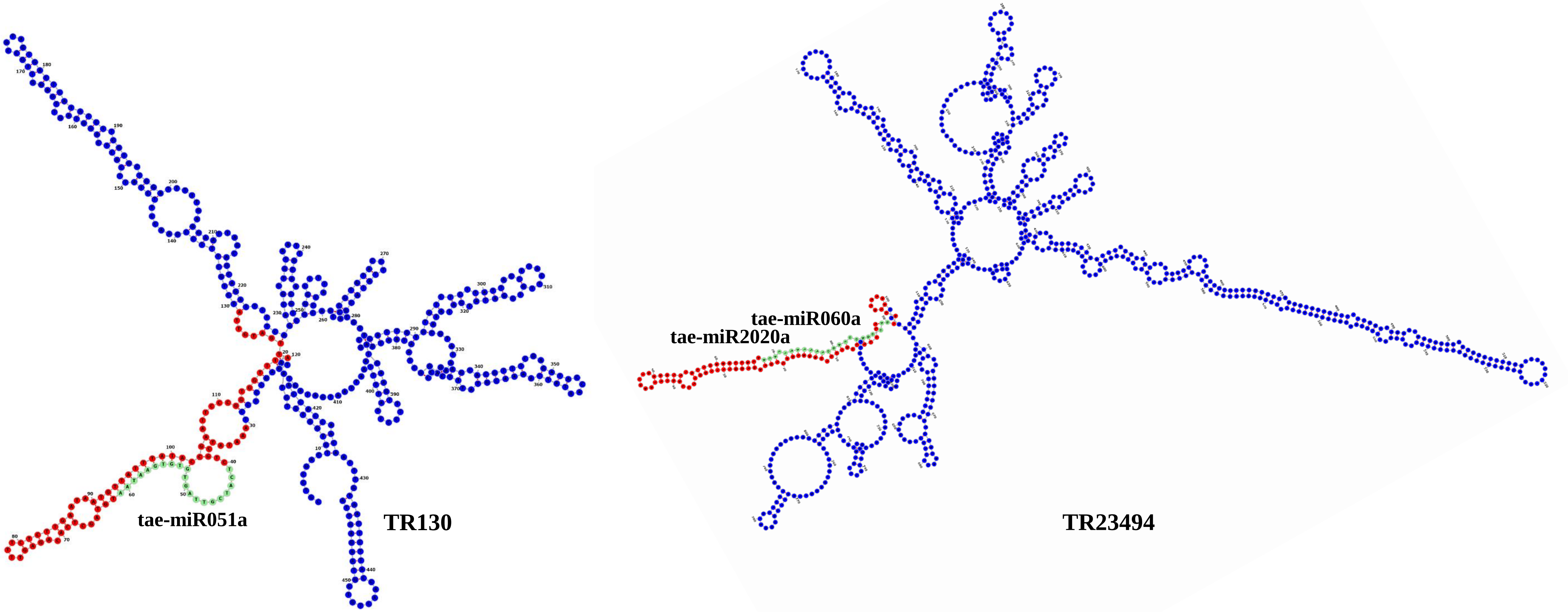
tae-miR051a
TR130
tae-miR060a
tae-miR2020a
TR23494

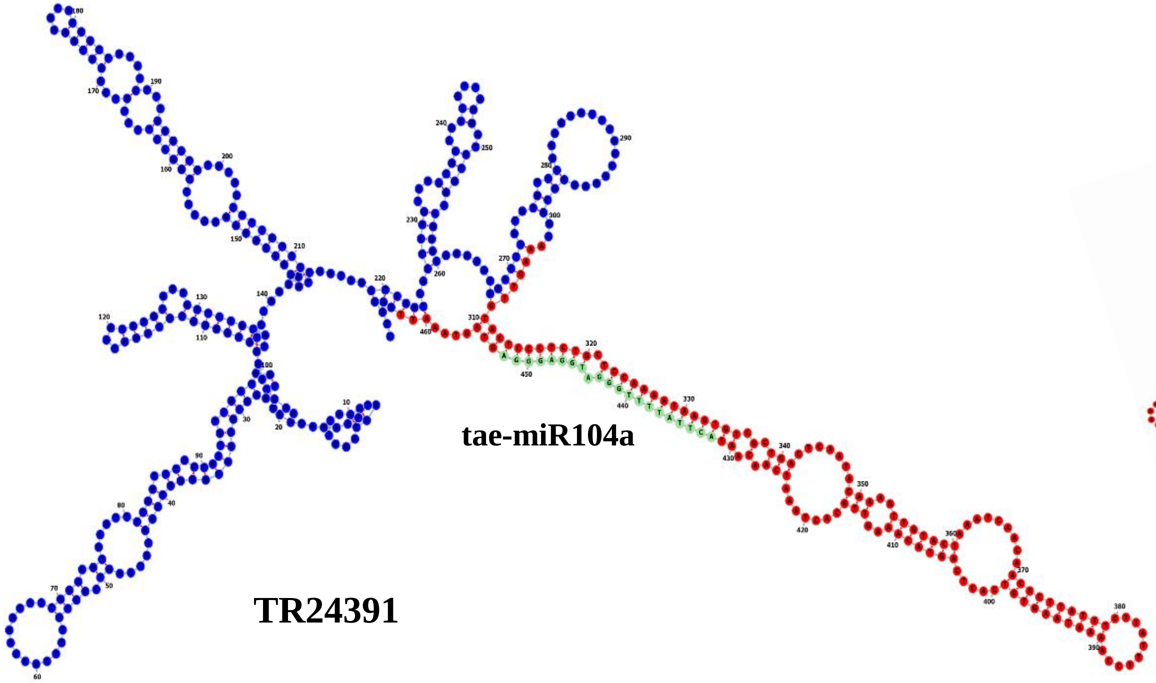

TR24391

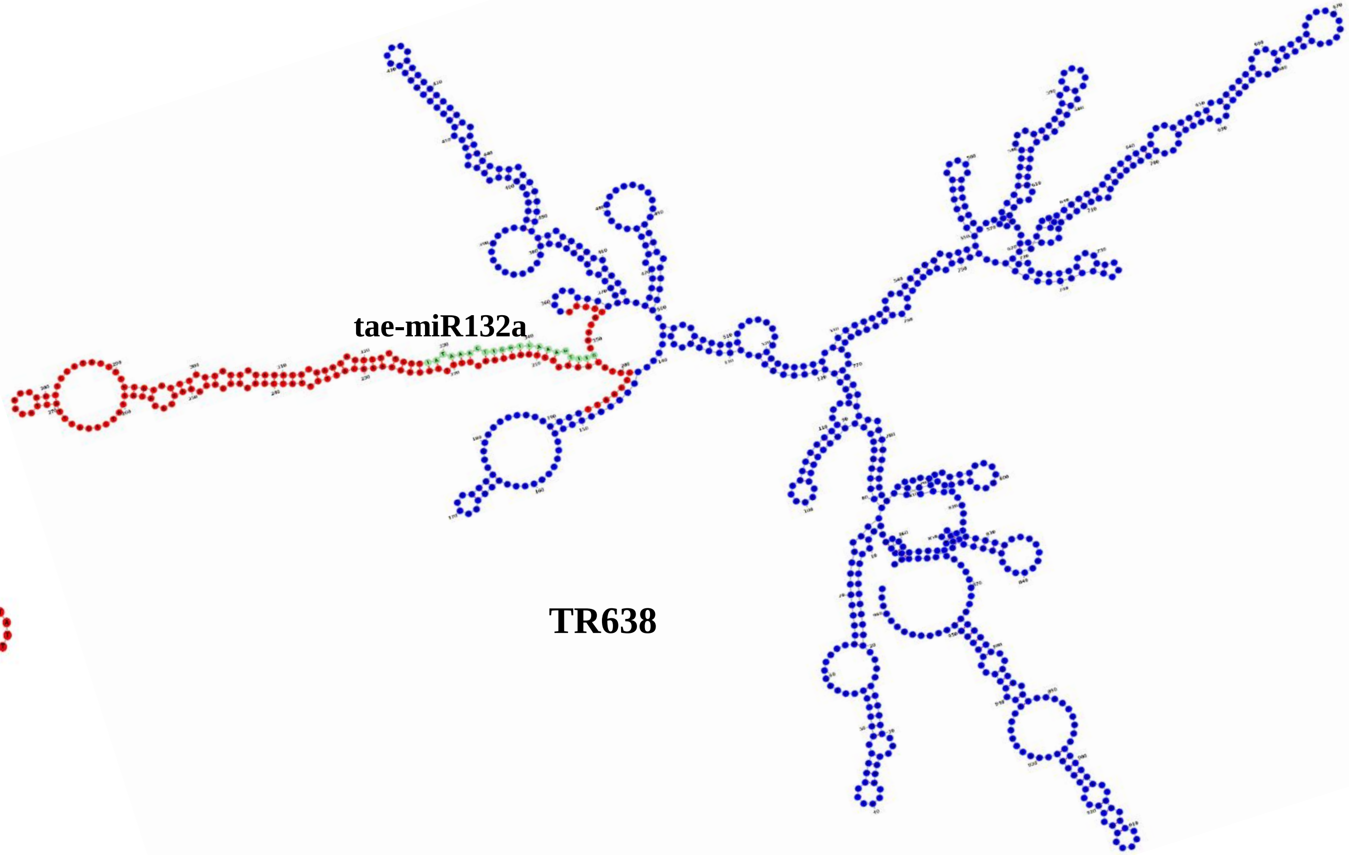

TR638

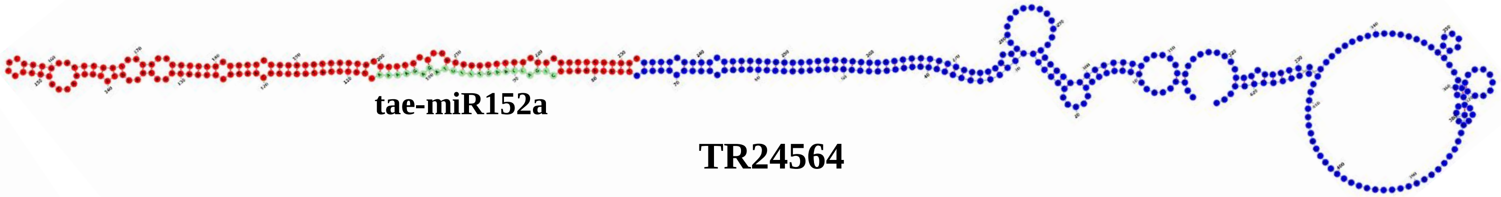
tae-miR152a
TR24564

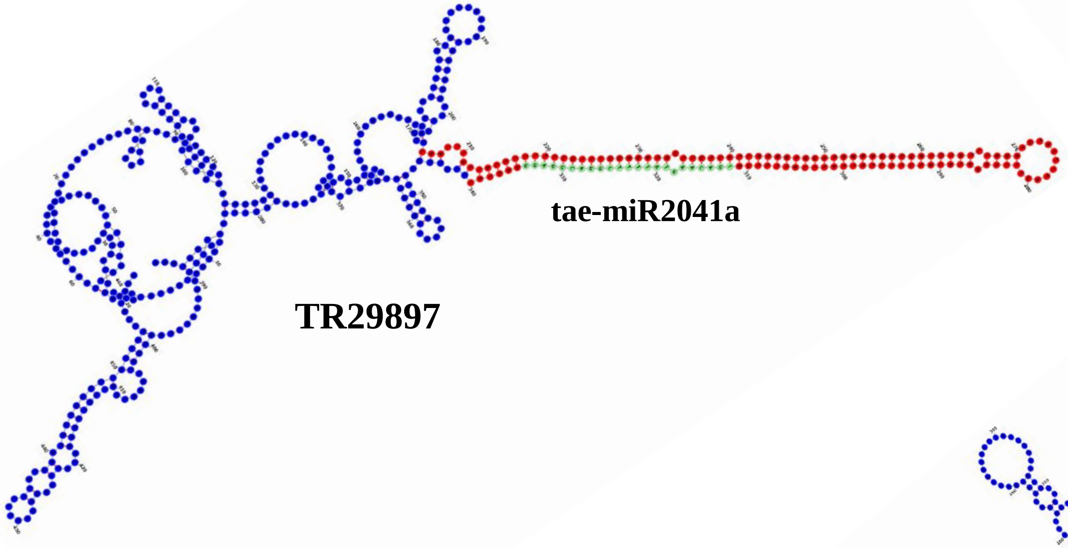
tae-miR2041a
TR29897

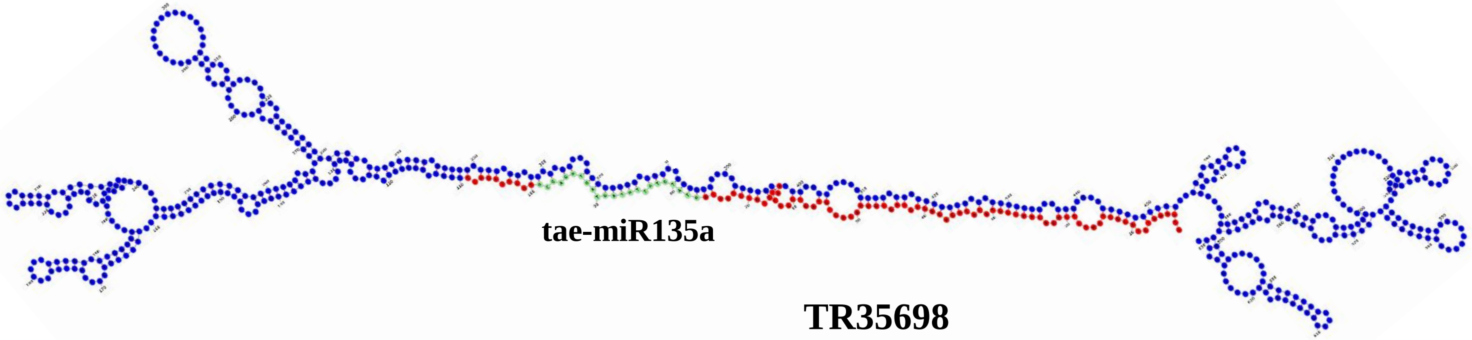
tae-miR135a
TR35698

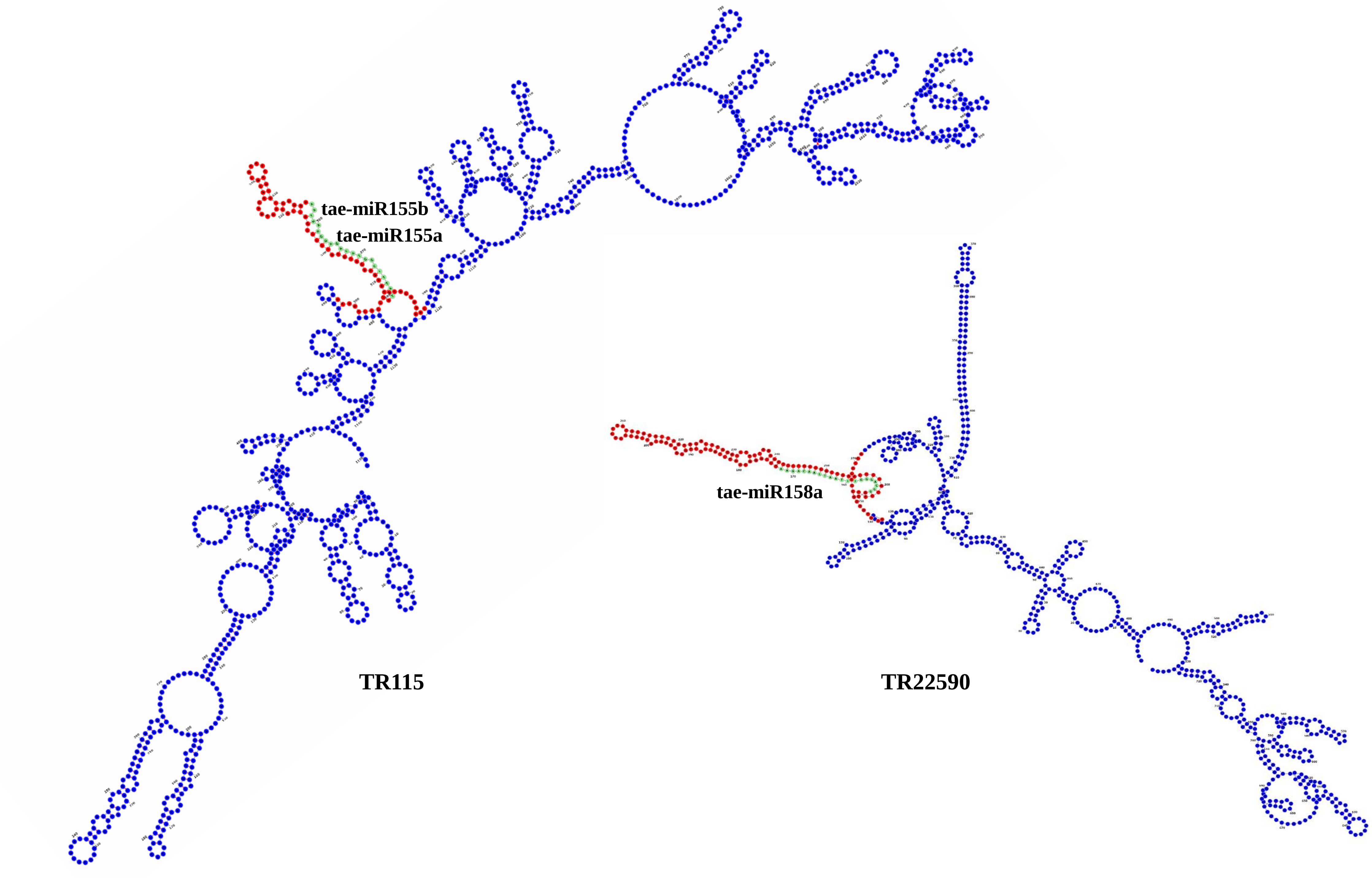
tae-miR155b
tae-miR155a
tae-miR158a
TR115
TR22590
