# Supplementary material for: Survey of High Throughput RNA-Seq Data Reveals Potential Roles for lncRNAs during Development and Stress Response in Bread Wheat
Source: Front Plant Sci. 2017 Jun 9;8:1019. doi: 10.3389/fpls.2017.01019 (PMC5465302; doi:10.3389/fpls.2017.01019)

Figure S3 Complete interaction network of lncRNAs with miRNAs and mRNAs. The lncRNA, miRNAs and mRNAs are represented in blue, orange and green circles, respectively.

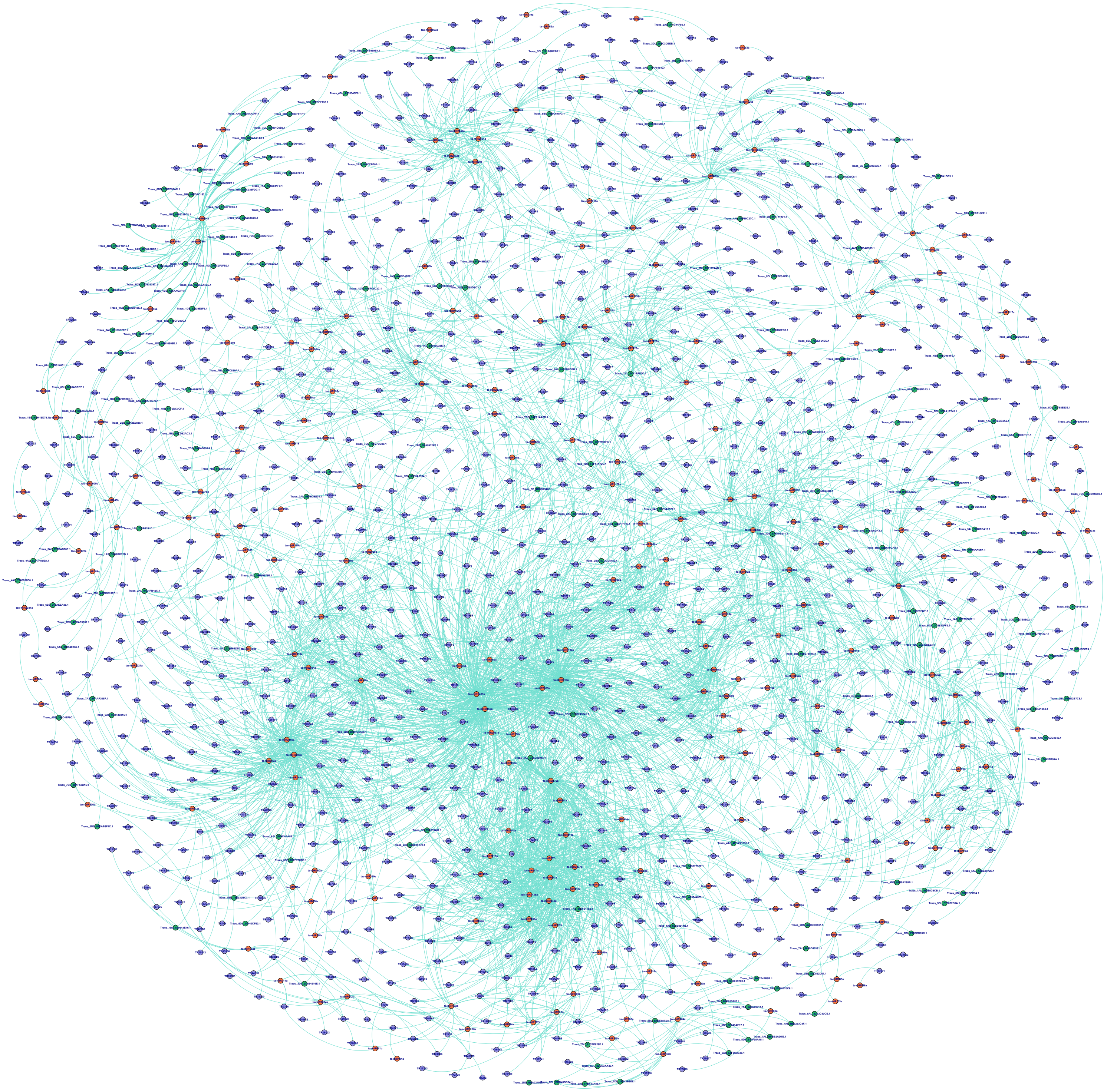

Supplement: Figure S3 — Complete interaction network of lncRNAs with miRNAs and mRNAs. The lncRNA, miRNAs and mRNAs are represented in blue, orange and green circles, respectively. [file Image3.PDF]
